# Self‐powered technology based on nanogenerators for biomedical applications

**DOI:** 10.1002/EXP.20210152

**Published:** 2021-09-01

**Authors:** Yuanzheng Zhang, Xiangyang Gao, Yonghui Wu, Jinzheng Gui, Shishang Guo, Haiwu Zheng, Zhong Lin Wang

**Affiliations:** ^1^ Key Laboratory of Artificial Micro‐ and Nano‐structures of Ministry of Education School of Physics and Technology Wuhan University Wuhan P. R. China; ^2^ International Joint Research Laboratory of New Energy Materials and Devices of Henan Province Henan University Kaifeng P. R. China; ^3^ Beijing Institute of Nanoenergy and Nanosystems Chinese Academy of Sciences Beijing P. R. China; ^4^ School of Materials Science and Engineering Georgia Institute of Technology Atlanta Georgia USA

**Keywords:** implantable, medical application, nanogenerators, physiological sensors, self‐powered, wearable

## Abstract

Biomedical electronic devices have enormous benefits for healthcare and quality of life. Still, the long‐term working of those devices remains a great challenge due to the short life and large volume of conventional batteries. Since the nanogenerators (NGs) invention, they have been widely used to convert various ambient mechanical energy sources into electrical energy. The self‐powered technology based on NGs is dedicated to harvesting ambient energy to supply electronic devices, which is an effective pathway to conquer the energy insufficiency of biomedical electronic devices. With the aid of this technology, it is expected to develop self‐powered biomedical electronic devices with advanced features and distinctive functions. The goal of this review is to summarize the existing self‐powered technologies based on NGs and then review the applications based on self‐powered technologies in the biomedical field during their rapid development in recent years, including two main directions. The first is the NGs as independent sensors to converts biomechanical energy and heat energy into electrical signals to reflect health information. The second direction is to use the electrical energy produced by NGs to stimulate biological tissues or powering biomedical devices for achieving the purpose of medical application. Eventually, we have analyzed and discussed the remaining challenges and perspectives of the field. We believe that the self‐powered technology based on NGs would advance the development of modern biomedical electronic devices.

## Graphical abstract

With the continuous development of self‐powered technology, self‐powered biomedical electronic devices have attracted extensive attention due to their unprecedented advantages.

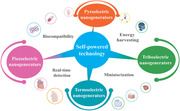

## 1 INTRODUCTION

Biomedical electronic devices have undergone rapidly developed in the past decades to enhance the quality of life and extend the life span of patients by providing physiological detecting and targeted therapy.^[^ ^1^, ^2^, ^3^, ^4^ ^]^ For example, since the first implantable pacemaker was invented in 1958, which has already saved millions of lives by preventing and treat life‐threatening cardiac conditions.^[^ ^5^ ^]^ Since then, immense improvements in biomedical electronic devices have been made.^[^ ^6^, ^7^, ^8^ ^]^ With the growth, general aging, and the extension of life expectancy, modern society has higher and higher requirements for biomedical electronic devices. However, some drawbacks curtail the rapidly developing and widespread application of biomedical electronic devices, primarily associated with limited battery life, lead pollution, miniaturization, and device‐related infections.^[^ ^9^ ^]^ For instance, conventional batteries have a limited life span. When the batteries are depleted, a new device will need to be replaced surgically, which will increase the risk of infection and death as well as the economic burden to the patients.^[^ ^10^, ^11^ ^]^ Therefore, an urgent need for a technique that can effectively solve these problems is a crucial factor in promoting the further development of biomedical electronic devices.

**FIGURE 1:**
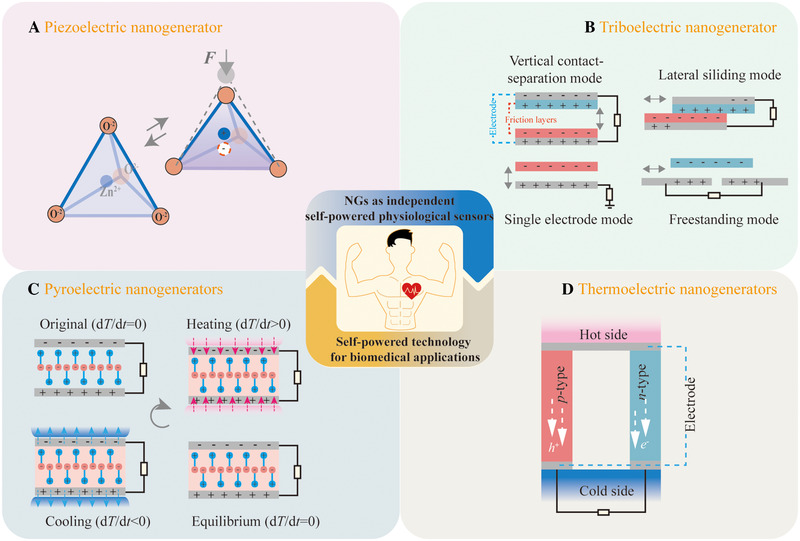
Schematic representation of the research contents based on NGs, including (A) PENG, (B) TENG, (C) PyNG, and (D) TEG

Since Wang's group firstly invented piezoelectric nanogenerator (PENG) and triboelectric nanogenerator (TENG) in 2006 and 2012, those two energy harvest techniques have become ideal choice for energy conversion.^[^ ^12^, ^13^ ^]^ The subsequent pyroelectric nanogenerators (PyNG) and thermoelectric nanogenerators (TEG) are also brilliant in harvesting waste heat.^[^ ^14^, ^15^, ^16^, ^17^ ^]^ At the same time, self‐powered technology aims to build self‐powered nanosystems that can harvest energy from the environment to power themselves.^[^ ^18^ ^]^ Recently, self‐powered technology based on NGs has drawn widespread interest in the biomedical field due to the rapid development of NGs in output performance, miniaturization, and good biocompatibility.^[^ ^19^, ^20^ ^]^ The NGs developed for self‐powered biomedical electronic devices (SPBEs) have made significant progress in the last few years. In some circumstances, the harvested electrical signals by nanogenerators can directly be used for active sensing of physiological parameters.^[^ ^21^, ^22^ ^]^ Meanwhile, those devices can convert mechanical energy and thermal energy from human bodies into electrical energy for the shortcomings of traditional biomedical electronic devices such as short battery life. At present, many types of SPBEs have been successfully manufactured, used to detect vital signs of the human body, or treat diseases. What is more exciting is that with the advantages of wide raw material selection and biocompatibility of NGs, new biomedical electronic devices are expected to solve lead pollution and patient infection of traditional devices. Although Xia et al. recently summarized the latest development of biomedical applications based on TENG, with the progress of preparation technology and the expansion of raw material selection, three other NGs have also made significant progress in the field of biomedical.^[^ ^23^ ^]^ Headings “NGs as independent self‐powered physiological sensors” and “Self‐powered technology for biomedical applications” in Figure 1 are the two parallel main lines of this manuscript, along which we summarize the applications of self‐powered technologies in healthcare.

Following this section, we first give a detailed description of the working fundamental and essential characteristics of the PENG TENG, PyNG, and TEG in Section 2 to further comprehend the self‐powered technology. Then Section 3 focuses on applying NGs as physiological sensors. Section 4 summarizes several major applications of NGs in the biomedical field, such as nerve stimulation, tissue repairing, and cell differentiation. Finally, we also discuss the remaining challenges and future perspectives for future SPBEs development.

## 2 SELF‐POWERED TECHNOLOGY BASED ON NANOGENERATORS

In this section, by emphasizing the working mechanism of each NGs, we summarize the Peng, Teng, PyNG, and TEG, as shown in Figure 1. Also, we introduced the self‐powered technology in detail, intending to comprehend the SPBEs further.

### 2.1 Nanogenerator

So far, NGs are divided into four basic types according to the working mechanisms, as shown in Figure 1. The first two types of NGs are PENG and TENG, mainly used to capture mechanical energy from the environment and human body, such as, wind energy, acoustic energy, walk, heart beating, and internal movement of organs.^[^ ^24^, ^25^, ^26^, ^27^, ^28^, ^29^ ^]^ Besides mechanical energy, heat is the other significant and valuable energy source in the environment. When the human body performs daily activities, a temperature difference can be formed between the body and the environment. As soon as a temperature gradient or temperature difference is created, the PyNG and TNG can effectively capture the wasted heat energy.^[^ ^30^, ^31^ ^]^ The working mechanisms of PENG, TENG, PyNG, and TEG will be described in detail in the following sections.

#### 2.1.1 Piezoelectric nanogenerators

When a mechanical stimulus is exerted to the piezoelectric materials, a piezoelectric potential will be generated in the direction of the external mechanical stimulus, as depicted in Figure 1A. Piezoelectric materials can generate continuous alternating pulsed electrical signals in response to dynamic external mechanical stimuli. PENG was invented based on the direct piezoelectric effect mentioned above.^[^ ^32^ ^]^ For biomedical applications, the selection of various biocompatible function materials, structure design, and packaging are the primary consideration issues.

Generally, the open‐circuit voltage (*V*_OC_) and short‐circuit current (*I*_SC_) of the flexible PENG can be expressed by the following equations^[^ ^33^, ^34^ ^]^

(1) $$V_{\mathrm{OC}}=k_{1}\times h\times \frac{d_{33}}{\varepsilon_{33}^{T}}\times \Delta \sigma$$

(2) $$I_{\mathrm{SC}}=k_{2}\times A\times \frac{\Delta \sigma }{\Delta t}\times d_{33}$$

where *k*_1_ and *k*_2_ are two different constants, *d*_33_ and *h* are the equivalent piezoelectric coefficient (C N^−1^) and thickness of the PENG, respectively. $\varepsilon_{33}^{T}$ is the permittivity at constant stress along the polarization direction, *A* is the area of the opposite faces, and Δσ is mechanical stress. It can be seen that the above equations describe the output characteristics of the PENG are related to the properties of the piezoelectric material and mechanical stress and have nothing to with the frequency of external compressive force. However, it should be noted that the accumulated charge generated during long‐time operation may affect the output performance.^[^ ^35^ ^]^

In 2006, the first PENG based on ZnO was invented by Wang and his co‐workers, indicating a new method of converting mechanical energy into electrical energy to drive nanodevices was created.^[^ ^13^ ^]^ In the past few years, PENG has made major breakthroughs in output performance, including *V*_OC_, *I*_SC_, and maximum output power density (*P*_d_). As shown in Table 1, we have summarized the performance comparison of various PENG recently reported.

**TABLE 1:** Output characteristics comparison of PENGs

		Performance			
Piezoelectric filler	Matrix	*V*_OC_ [V]	*J*_SC_ [μA cm^−2^]	*P*_d_ [μW cm^−2^]	Ref.
Pb(Mg_1/3_Nb_2/3_)O_3_‐PbTiO_3_	PDMS	60	0.85	11.5	[36]
0.91K_0.48_Na_0.52_NbO_3_–0.04Bi_0.5_Na_0.5_ZrO_3_–0.05AgSbO_3_–0.2% Fe_2_O_3_	PDMS	52	1.2	3.62	[20]
0.82Ba(Ti_0.89_Sn_0.11_)O_3_–0.18(Ba_0.7_Ca_0.3_)TiO_3_	PDMS	39	0.42	6.05	[22]
Bi_0.84_Sm_0.16_Fe_0.84_Ti_0.16_O_3_	Silicon rubber	16	0.62	3.11	[19]
P(VDF‐TrFE)	N/A	6	2	1.4	[37]
P(VDF‐TrFE)/GeSe	N/A	5.27	0.38	9.76	[38]
GaN	N/A	19.7	1.9	39	[39]
PMN‐PT	PDMS	105	290	N/A	[40]
BiCl_3_/PVDF	N/A	38	0.88	0.2	[41]
P(VDF‐TrFE)	N/A	150	0.1	8.75	[42]
0.5(Ba_0.7_Ca_0.3_)TiO_3_−0.5Ba(Zr_0.2_Ti_0.8_)O_3_	PDMS	23	0.35	2	[43]
0.915(Na_0.5_K_0.5_)(Nb_0.94_Sb_0.06_)O_3_–0.045LiTaO_3_–0.04BaZrO_3_	PDMS	18	6.5	N/A	[44]
P(VDF‐TrFE)/MgO	N/A	2	N/A	N/A	[25]
P(VDF‐TrFE)	N/A	9.3	0.19	1.76	[45]
P(VDF‐TrFE)/BaTiO_3_/graphene	N/A	11	N/A	0.65	[46]
P(VDF‐TrFE)	N/A	2.7	0.1	0.8	[47]
P(VDF‐TrFE)/BaTiO_3_/Ag nanowires	N/A	14	0.24	0.986	[48]
PbZr_0.52_Ti_0.48_O_3_	Silicon rubber	20	0.02	3.93	[49]
PVDF/BaTiO_3_/n‐type graphene	N/A	10	0.31	0.72	[50]
Cellulose nanofibril/PVDF	N/A	60.2	5.1	6.3 mW cm^−2^	[51]
BaTiO_3_/bacterial cellulose	N/A	14	0.19	0.64	[52]
BiFeO_3_	PDMS	3	0.12	N/A	[53]
PVDF/AlO/graphene	N/A	36	0.8 μA	27.97	[54]
BaTi_(1−_ *_x_*_)_Zr*_x_* O_3_/PVDF	N/A	11.9	1.39 μA	0.14	[55]
P(VDF‐TrFE)	N/A	7	58 nA	0.56	[34]
Ba_0.85_Ca_0.15_Zr_0.10_Ti_0.90_O_3_/polydopamine	Polylactic acid	14.4	0.28	7.54 mW cm^−3^	[56]
BaTiO_3_/carbon tubes	PDMS	4.6	1.84 μA	8.8	[57]
Ba_1−_ *_x_* La_x_Sn_0.09_Ti_0.91_O_3_	PDMS	50	0.6 μA	48 mW cm^−2^	[58]
Graphene quantum dot/PVDF	N/A	6	25 nA	N/A	[59]
P(VDF‐TrFE)/ZnO	N/A	61	2.2 μA	N/A	[60]

#### 2.1.2 Triboelectric nanogenerators

Generally, the TENGs are classified into four types of working modes according to the characteristics of the structure, as shown in Figure 1B.^[^ ^61^ ^]^ The energy capture ability of the TENG relies on the coupling between the triboelectrification effect and electrostatic induction.^[^ ^62^ ^]^ When two different friction materials are in contact, the frictional charge moves to both surfaces due to the triboelectrification effect.^[^ ^63^ ^]^ When the friction materials are separated, a potential difference will appear between the two. It changes with the contact and separation of friction materials surfaces. Therefore, the external free electrons will migrate to screen the potential difference, resulting in an electrical signal. The output performance of the TENG can be calculated as the below equations^[^ ^28^, ^64^ ^]^

(3) $$V_{\mathrm{OC}}=\frac{Qd}{S\varepsilon }$$

(4) $$I_{\mathrm{SC}}=\frac{dQ}{dt}$$

(5) $$Q_{\mathrm{SC}}=\int I_{SC}dt$$

where *Q* is the transfer charges, *d* is the sliding displacement, *S* is the area of the opposite faces, ε is the permittivity, and *t* is the costed time during a cycle.

To improve the practicality of TENG, various methods such as the shuttling of charges and inductively coupled plasma (ICP) etching are used to improve the output characteristics.^[^ ^6^, ^12^, ^65^ ^]^ At the same time, more and more polymers choose to be used as friction materials to prepare high‐performance TENG, such as polydimethylsiloxane (PDMS), polyethy (PET), polytetrafluoroethylene (PTFE), polymethyl methacrylate (PMMA), polyamide (PA), polyvinyl alcohol (PVA), and fluorinated ethylene propylene (FEP). The performance comparison of various TENGs recently reported is listed and shown in Table 2.

**TABLE 2:** Output performance comparison of TENGs

	Performance			
Friction materials	*V*_OC_ [V]	*J*_SC_ [μA cm^−2^]	*P*_d_ [W m^−2^]	Ref.
PTFE/PMMA	530	10.5 μA	1.5 mW	[28]
PVDF/Kapton	400	1.14	7	[66]
PTFE/PET	1500	0.43	1.18	[67]
Kapton/PET	191	N/A	8.75	[68]
PTFE/Arcylic	3750	28 μA	2.4	[69]
PA/ cellulose nanofibrils	35	0.189	1.35	[70]
FEP/Al	75	75 μA	0.5	[71]
PVDF/PVA	230	1.5	3.1	[72]
PDMS/Cu	57	0.33	0.06	[73]
FEP/Cu	N/A	N/A	244	[74]
PDMS/human skin	1000	2.6	0.5	[75]
PDMS/Al	465	26.87	125	[76]
PTFE/Nylon	1300	0.41	5.3	[77]
PDMS/Cu	200	0.31	4.36	[78]
PDMS/Al	148	0.32	0.63	[79]
PVDF/ Poly(3‐hydroxybutyrate‐*co*‐3‐hydroxyvalerate)	695	2.9	3.1	[80]
PVDF/rubber	2209	48	56.9	[81]
PDMS/TPU‐Ag nanowires	95	0.01	0.61 μW cm^−2^	[82]
PBOA/ poly(ethylene oxide)	40	0.08	2.8 μW cm^−2^	[83]
FEP/aerogel	105.6	5.01	560	[84]
PVDF/cellulose	106.2	4.6	13.3	[85]
PDMS/hybrid cryogel	262.14	13.76	7.44	[86]
Liquid crystal polymer/Cu	1000	10.1	12.4	[87]
PTFE/Cu	2450	2.2	N/A	[88]
PTFE/PDMS	275	0.98	0.8	[89]
PDMS/PVA	270.2	0.44	N/A	[90]
PVDF/PET	1140	9.2	N/A	[91]
PTFE/Al	1290	180	N/A	[83]
PP/Zn	850	N/A	25.8	[65]
PTFE/PMMA	233	3.18	N/A	[92]
FEP/Acrylic	1670	13.4 μA	0.016	[93]

#### 2.1.3 Thermoelectric nanogenerators

A distinctive feature of TEG is that it collects the surrounding thermal energy and converts it into electrical energy through the Seebeck effect. The working principle of TEG relies on the migration of carriers (holes in the p‐type and electrons in the n‐type) within the thermoelement pair. When TEG is in a temperature gradient environment, holes and electrons spread from the hot side to the cold side, causing a potential difference due to the accumulation of the carries in the cold side.^[^ ^94^ ^]^ Figure 1C depicts the working principle of TEGs. The efficiency of TEG is determined by the dimensionless figure of merit of thermoelectric material, which can be specified as

(6) $$\mathrm{ZT}=\frac{S^{2}\sigma T}{k_{e}+k_{\mathrm{lat}}}$$

where

(7) $$k_{e}=\;neLT\mu$$

where *σ is* electrical conductivity, *S* is the Seebeck coefficient of the thermoelectric materials, *k*_e_ represents the thermal conductivity of the electron, *k*_lat_ is the lattice thermal conductivity, and *T* is the absolute temperature; *L*, *e*, *n*, and μ define Lorenz number, electrical charge carrier, carrier concentration, and carrier mobility, respectively. Thus, thermoelectric material with high *S* and *σ* is required to obtain a high electrical energy conversion rate.

Optimization via doping and nanostructures has an essential effect on the output performance of the TEGs. In 2012, Yang et al. demonstrate a TEG made from Sb‐doped ZnO nanobelt. When the TEG was bonded on a glass substrate under a temperature difference of 30 K, it can produce a voltage and current of 0.01 V and 194 nA, respectively. The Seebeck coefficient of the device reaches −350 μV/K.^[^ ^14^ ^]^ Subsequently, a flexible TEG based on a Te‐nanowire/poly(3‐hexylthiophene) (P3HT) polymer composite has been designed. When the temperature difference is 55 K, two TEGs in series can produce a voltage of 0.038 V.^[^ ^95^ ^]^ Although TEG application has been expanding steadily in recent years, limited by its working principle and energy conversion efficiency, the medical applications related to TEG are rarely reported. In the future, with the development of high Seebeck coefficient materials and preparation technology, TEG may play an essential role in the Internet of Things (IoT) and healthcare.

#### 2.1.4 Pyroelectric nanogenerators

The TEG cannot work when the environment temperature is spatially uniform and the temperature varies with time. In these circumstances, PyNGs have become the main means of collecting heat energy.^[^ ^96^ ^]^ The pyroelectric effect is caused by the change of spontaneous polarization in anisotropic materials caused by temperature fluctuation. When the temperature is unchanged, no pyroelectric current is produced due to the spontaneous polarization intensity of the materials remains constant.^[^ ^97^ ^]^ Once the temperature fluctuates, the spontaneous polarization intensity of the materials will be changed. Therefore, the pyroelectric current is yield in the external circuit. Figure 1C shows the working mechanism of the PyNGs. According to the pyroelectric theory, the potential *U* between the two electrodes can be acquired by the following equations^[^ ^98^ ^]^

(8) $$U=Ed=\frac{\Delta P\times \Delta T}{\left(\varepsilon_{r}-1\right)\varepsilon_{0}}$$

where ΔT is the change in the temperature, ΔP is the change of the polarization within the sample, $\varepsilon_{r}$ is the relative dielectric constant, $\varepsilon_{0}$ is the permittivity of the vacuum dielectric constant.

In 2012, a PyNG based on PZT for monitoring temperature change was proposed. Unlike the TEG, the output voltage of the PyNG will increase linearly with the rate of temperature change. The minimum detection limit of temperature changes at room temperature is about 0.4 K. Meanwhile, the self‐powered temperature sensor's response time and reset time is about 0.9 and 3 s, respectively.^[^ ^15^ ^]^ Afterward, Yang et al. have been fabricated a flexible PyNG based on KNbO_3_ nanowires. In this work, the PyNG was also used to harvest energy from sunlight illumination. When the temperature‐induced by sunlight illumination periodically changes from 295 to 298 K, the devices exhibit optimal output voltage and current of 2.5 mV and 25 pA, respectively.^[^ ^17^ ^]^ So far, PyNG‐based self‐powered temperature sensors have potential applications for temperature measurement in the IoT, medical diagnostics, and other fields.

### 2.2 Commonalities and differences between four types of nanogenerators

In summary, four types of NGs can convert energy from the environment into electricity. Among them, PENG is the first one invented among the four, and the flexible PENG has attracted wide attention. In order to improve the output performance of PENG, various advanced preparation processes and materials with high piezoelectric coefficients have been widely adopted, such as, photolithography and KNN‐based piezoelectric materials. In addition to harvesting mechanical energy from the environment, the all‐in‐one PENG is particularly suitable as an implantable biomedical electronics. Subsequently, TENG, invented in 2012, accelerated the progress of self‐powered technology with its wide selection of materials and high output performance.^[^ ^12^ ^]^ Due to the limitation of the operating principle, the structure of TENG needs to retain a small gap for contact separation motion, so further efforts are needed in the miniaturization of the device. On the other hand, miniaturization of the device affects the output performance of the TENG, so finding a balance between the two is the key to promote TENG in medical applications.

Thermal energy is another widely available energy in the environment besides mechanical energy, and the differences between TEG and PyNG, which can convert thermal energy from the environment to electrical energy, have been discussed above. However, limited by the working principle and conversion efficiency, the application of both in medical applications has only been reported for respiratory sensors, so the application in medical applications still needs to be further explored.

### 2.3 Self‐powered technology

Developing self‐powered devices with battery‐free and long‐term independent working is critical for defense technology, IoT, and even healthcare. In biomedical, wearable and implantable electronic devices need to be miniaturized and have a long life, but the two are contradictory. The small size of the battery, in turn, limits the life of the device. Hence, an urgent need for technology makes wearable or implantable biomedical devices self‐powered without a battery. This technology can ameliorate the adaptability of devices and significantly reduce their size and weight. In 2005, Wang's group proposed a self‐powered technology based on NGs that aim to build self‐powered systems with ultra‐small size, super sensitivity, extraordinary multifunctionality, and extremely lower power consumption is expected to solve this problem well.^[^ ^18^, ^99^ ^]^ Therefore, such NGs‐based electronic devices with the above‐mentioned characteristics are widely considered as self‐powered devices. The SPBEs can harvest energy from the environment to power themselves, meet the requirements that continuously and accurately monitored medical signals. With this technology, biomedical electronic devices will have enormous benefits for healthcare and quality of life.

The application examples discussed in this manuscript are all summarized on this basis. They are all used in practical applications to convert mechanical and biomechanical energy into electrical energy, detecting important physiological information in the human body, or stimulating tissues for medical applications. Therefore, we discuss the two main lines of self‐powered sensors (Section 3) and self‐powered technologies for medical applications (Section 4).

## 3 NANOGENERATORS AS SELF‐POWERED PHYSIOLOGICAL SENSORS

**FIGURE 2:**
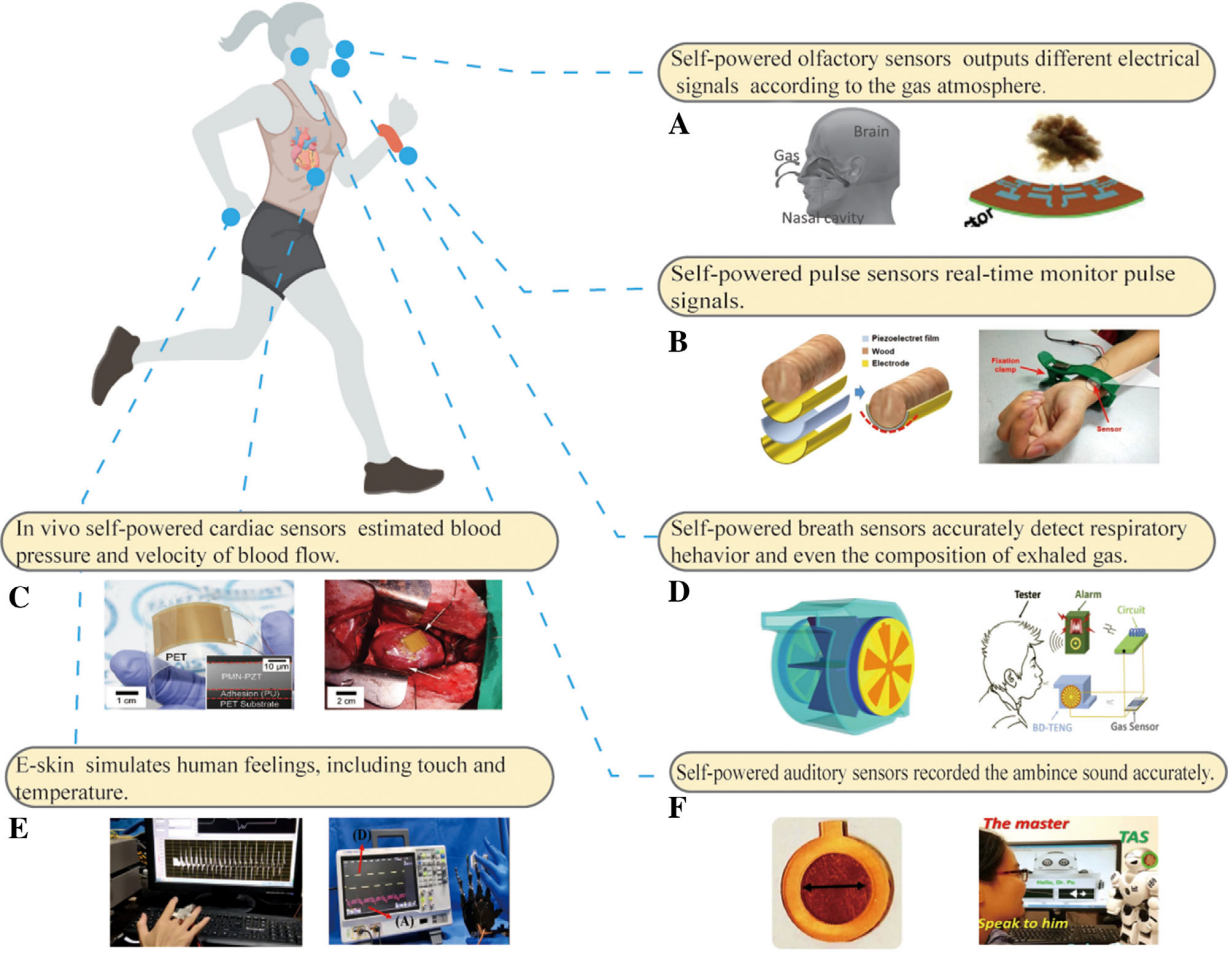
NGs as physiological sensors are used in the biomedical field with examples. (A) Self‐powered olfactory sensors. Reproduced with permission.^[^ ^100^ ^]^ Copyright 2019, Elsevier. (B) Self‐powered pulse sensors. Reproduced with permission.^[^ ^103^ ^]^ Copyright 2019, Elsevier. (C) In vivo self‐powered cardiac sensors. Reproduced with permission.^[^ ^29^ ^]^ Copyright 2017, Wiley‐VCH. (D) Self‐powered breath sensors. Reproduced with permission.^[^ ^101^ ^]^ Copyright 2015, Elsevier. (E) Electronic skim. Reproduced with permission.^[^ ^22^ ^]^ Copyright 2021, Elsevier. (F) Self‐powered auditory sensors. Reproduced with permission.^[^ ^102^ ^]^ Copyright 2018, American Association for the Advancement of Science

In this review, SPBEs are divided into two categories according to their different working methods. The first category is that NGs act as independent sensors, directly converting the biomechanical energy and heat energy produced by physiological activities into electric signals. The physiological sensor based on NGs can accurately and real‐time monitor vital signals by analyzing the output signals. Herein, the typical physiological sensors are reviewed and introduced in detail based on practical applications, such as cardiac/pulse sensors, artificial sensory organs, and breath sensors, as presented in Figure 2.

### 3.1 Self‐powered cardiac and pulse sensors

Changes in pathological and physiological parameters are of great importance in clinical practice. Failure to monitor sudden changes in vital signs on time can often lead to death in a short period. Therefore, plenty of implantable, wearable, miniaturized physiological sensors based on NGs have been developed to prevent missing this momentous medical information.

**FIGURE 3:**
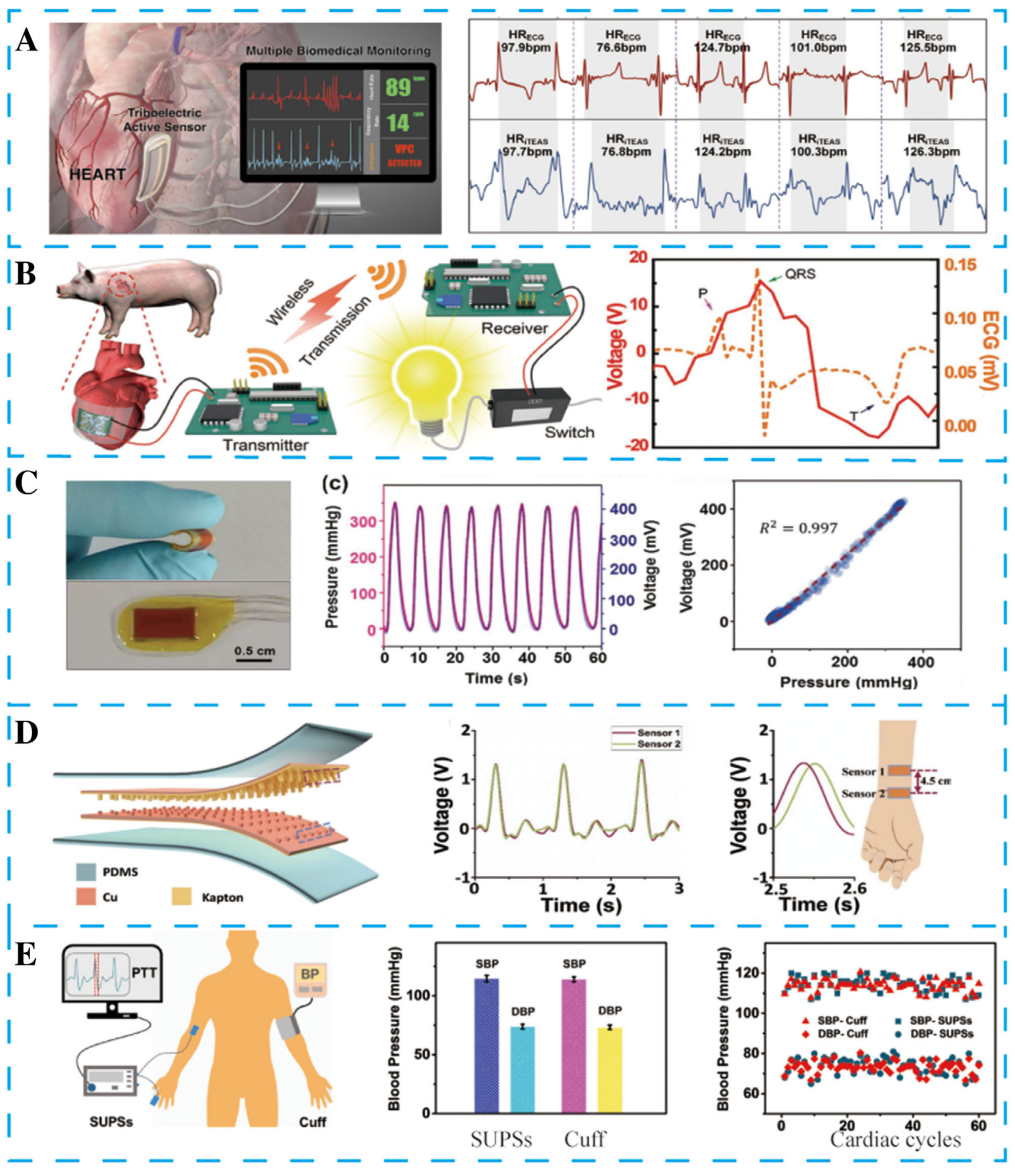
Self‐powered cardiac and pulse sensors based on NGs. (A) Implantable self‐powered cardiac sensor for real‐time heart rates and blood pressure monitoring. Reproduced with permission.^[^ ^104^ ^]^ Copyright 2016, American Chemical Society. (B) In vivo biocompatible flexible energy harvester for self‐powered wireless transmission. Reproduced with permission.^[^ ^29^ ^]^ Copyright 2017, Wiley‐VCH. (C) Transcatheter self‐powered ultrasensitive endocardial pressure sensor based on TENG. Reproduced with permission.^[^ ^23^ ^]^ Copyright 2019, Wiley‐VCH. (D) Flexible self‐powered pulse sensor based on TENG. Reproduced with permission.^[^ ^105^ ^]^ Copyright 2017, Wiley‐VCH. (E) Self‐powered ultrasensitive pulse sensor based on TENG for noninvasive multi‐indicators cardiovascular monitoring. Reproduced with permission.^[^ ^106^ ^]^ Copyright 2021, Elsevier

In 2016, Ma et al. proposed a flexible implantable cardiac senor based on TENG, providing accurate, continuous, and real‐time monitor of vital signs (Figure 3A).^[^ ^104^ ^]^ The primary friction materials of implantable triboelectric active sensor consist of nanostructured PTFE and Kapton film. The unique nanostructure and flexible packaging enable the sensor to monitor tiny changes of organs and obtain excellent durability and biocompatibility. An electric output with a *V*_OC_ of ∼10 V and an *I*_SC_ of ∼4 μA is obtained when the triboelectric active sensor is fixed to the pericardium of the living porcine model. At the same time, the peak value of the voltage signal is highly matched with the corresponding R wave in the electrocardiogram results. Furthermore, the blood pressure and velocity of blood flow could be estimated.

It is essential for implantable sensors to use the energy collected in the body for wireless data transmission. Kim et al. proposed a self‐powered wireless data transmission system driven by a high‐performance implantable PENG in a porcine model (Figure 3B). The polished PMN‐PZT‐Mn film is fixed on a PET without any waviness, fracture, or crack. Excellent crystallinity and reasonable structural design make this energy harvester have superior output performance. The PENG based on PMN‐PZT produces a *V*_OC_ of 17.8 V and an *I*_SC_ of 1.74 μA from porcine heartbeats, enabling the self‐powered drive system to transmit communication data wirelessly. Furthermore, when the capacitor connected with PENG was charged above the driving threshold voltage, a signal is sent to control the distant appliance. Although this flexible PENG is prepared using lead‐based piezoelectric materials, the self‐powered system has good biocompatibility after elaborate packaging, which means that the self‐powered sensor can replace the traditional implanted biomedical sensor.

As a critical factor for evaluating cardiac function, endocardial pressure has important clinical significance. In 2019, Liu et al. presented a self‐powered endocardial pressure sensor based on TENG (Figure 3C).^[^ ^23^ ^]^ In order to achieve ideal sealing, high sensitivity, and flexibility, a multilayer structure is introduced to prepare TENG. The overall size of the prepared TENG was 1 cm × 1.5 cm × 0.1 cm. After corona discharge treatment, the *V*_OC_ of TENG is increased five times to 6.2 V. The flexible TENG exhibits outstanding durability. In this study, a 2 cm skin incision was enough to implant the device, and the concomitant iatrogenic orthopedic injury was negligible. The output voltage signals of TENG are produced by the periodic changes of cardiac contraction and relaxation, so it carries abundant physiological parameters information. The self‐powered endocardial pressure sensor responds well over a wide range of pressure environments. Furthermore, this sensor can also detect cardiac arrhythmias such as ventricular premature contraction and ventricular fibrillation.

In 2017, Han et al. proposed a wearable ultrasensitive pulse sensor (SUPS) based on TENG with a high signal‐noise ratio (45 dB) and long‐term durability (10^7^ cycles) (Figure 3D).^[^ ^105^ ^]^ The entire sensor was encapsulated by a PDMS layer to guarantee its stability. This sensor can accurately, wireless, and real‐time monitor pulse signals of humans and transmit data to a smartphone or PC by a Bluetooth chip. When this sensor was fixed on the radial arteria, the peak waves of the detected signal are coordinated with the corresponding R wave in the electrocardiograph. The feature points and second derivative detected by the commercial pulse sensor were strictly following SUPS. Moreover, the sensitivity and accuracy of SUPS are comparable to that of commercial pulse sensors. The output signal can be transmitted wirelessly through a homemade wireless data transmission system. Finally, the physical health of volunteer patients can be accurately determined by analyzing the characteristic exponent of the pulse signal, such as pulse wave velocity.

Multi‐indicators cardiovascular real‐time monitoring is a significant challenge in clinical diagnosis. In 2021, Xu et al. proposed a SUPS based on TENG for noninvasive multi‐indicators cardiovascular monitoring (Figure 3E).^[^ ^106^ ^]^ FEP film and PA film acted as friction materials of the TENG. Cu copper was tapped to the backside of the friction layer was selected as the electrode. Sensors distributed throughout the body are integrated into a real‐time and sustained monitoring network. Leveraging the synergy between the material surface and the unique device structure, the sensitivity and response time of the SUPS as high as 10.29 nA kPa^−1^ and 30 ms, respectively. Various cardiovascular indicators, including blood pressure, heart rate, and pulse wave velocity, can be extracted from the detected pulse waveform by integrating SUPS distributed at different arterial pulse positions. All detection indicators are highly compatible with the testing results of commercial medical devices.

### 3.2 Self‐powered breath sensors

Respiration, as one of the primary activities of the human body, plays a crucial role in maintaining normal physiological signs. Therefore, continuous detecting and real‐time analyzing respiratory rhythm are enormously significant for patients' health management. Various self‐powered, portable, and miniaturized breath sensors based on NGs have been developed in past years to meet this goal.^[^ ^101^, ^112^, ^113^ ^]^ Those devices can effectively diagnose patients' internal diseases by monitoring respiratory rhythm and exhaled gas, which has great clinical significance in diagnosing and treating many diseases.

**FIGURE 4:**
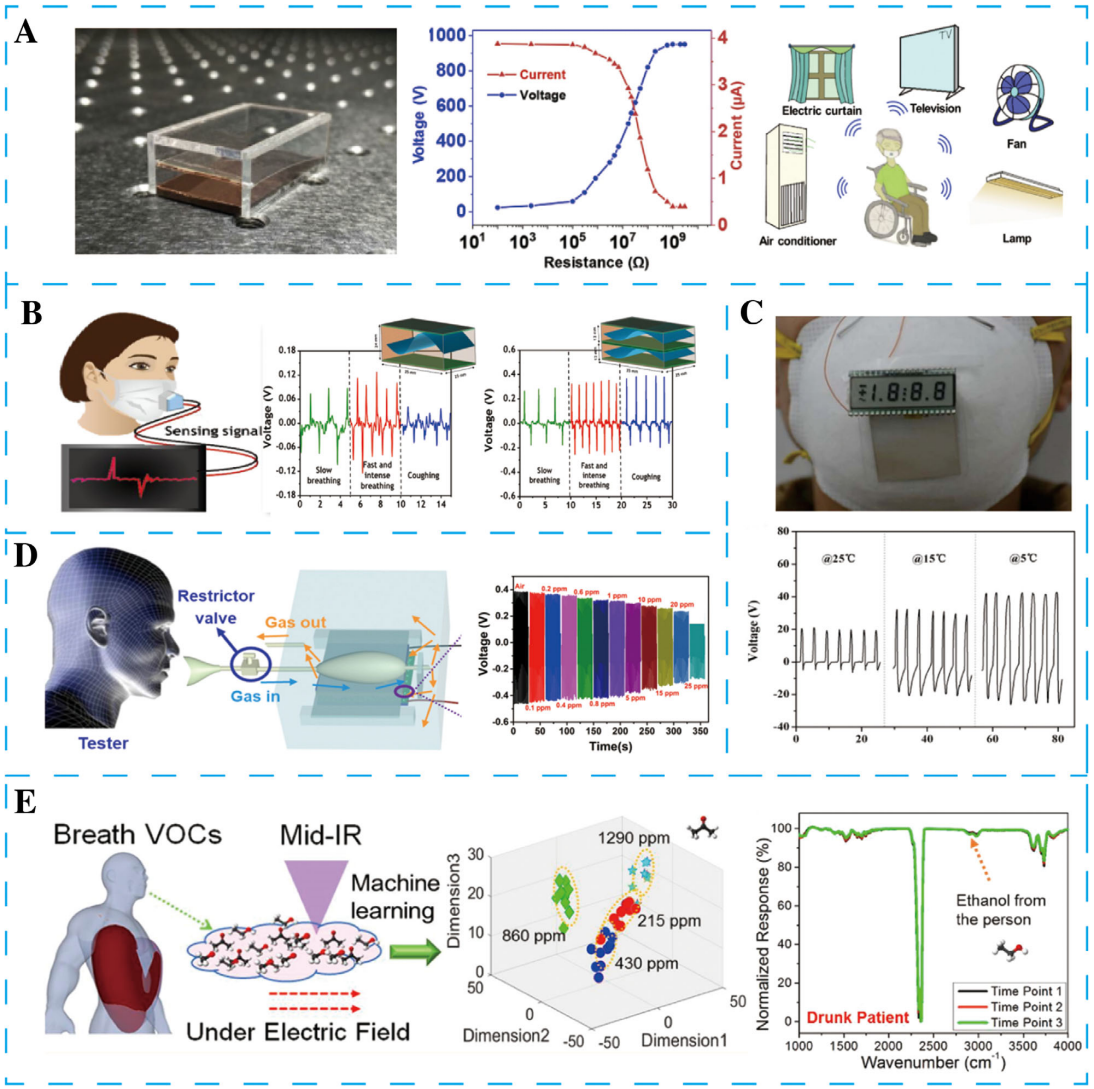
The application of NGs for self‐powered breath sensors. (A) Breath‐driven single‐electrode TENG for the human‐machine interface interaction. Reproduced with permission.^[^ ^107^ ^]^ Copyright 2019, Elsevier. (B) Biocompatible biomaterial‐based TENG for self‐powered breath sensor. Reproduced with permission.^[^ ^108^ ^]^ Copyright 2021, American Chemical Society. (C) Self‐powered human breathing and temperature sensors based on wearable PyNG. Reproduced with permission.^[^ ^109^ ^]^ Copyright 2017, Elsevier. (D) Respiration‐driven TENG for disease screening through multifunctional respiratory monitoring. Reproduced with permission.^[^ ^110^ ^]^ Copyright 2019, Elsevier. (E) Multiswitched manipulated TENG based on machine learning for volatile organic compound identification. Reproduced with permission.^[^ ^111^ ^]^ Copyright 2021, American Chemical Society

Existing traditional human–machine interfaces (HMI) are not suitable for some disabilities due to the difficulty expressing their intentions clear with touch and language. In 2019, Zhang et al. reported a breath‐driven sensor based on TENG to transmit control commands through breathing for the HMI (Figure 4A).^[^ ^107^ ^]^ The TENG device's relative size and total weights are 0.5 cm × 2 cm × 3.5 cm and 2.47 g, respectively. Such a portable device is convenient for the disabled to carry and use. As the airflow passes through the pipe, the vibration of the PET film makes cyclical contact separation with the electrode and generates electrical energy. Simultaneously, the device can generate different electrical signals according to different air velocity, and identify the standard and deliberately enhanced breathing. For example, the deliberately enhanced breath produces a stronger electrical signal than normal breathing, with output voltage and current reaching 342 V and 2.3 μA, respectively. Eventually, this HMI system based on this self‐powered sensor can convert biomechanical from human breathing into command signals to control electrical applications.

In 2021, Araz et al. reported a cellulose nanofibril‐based TENG using diatom bio‐silica to improve the output characteristics while maintaining the biocompatible function, as shown in Figure 4B.^[^ ^108^ ^]^ The biocompatible film and PTFE are used as two friction materials, respectively. Compared with cellulose, cellulose nanofibers have the advantages of transparency, robustness, flexibility, and excellent triboelectric performance. The enhanced output performance of the TENG is attributed to the SiO_2_ is the most positive material in the triboelectric series. As mentioned earlier, its the potential to improve triboelectric output. The TENG can generate *V*_OC_ and *I*_SC_ as high as 88 V and 18.6 μA under a constant force of 8 N, respectively. The smart mask can generate a maximum voltage of around 0.08 and 0.12 V according to slow breathing and fast (intense) breathing.

Xue et al. designed a wearable PyNG using PVDF thin film integrated into an N95 mask to harvest the energy produced by human breath (Figure 4C).^[^ ^109^ ^]^ The PyNG is consists of three layers, including PVDF film and two Al film electrodes. All the experimental steps were performed at 5°C to test the performance of the PyNGs. When the PyNG is installed on the mask at the location where the airflow of the breath is most concentrated, the temperature fluctuation caused by human respiration could produce a *V*_OC_ of 42 V and an *I*_SC_ of 2.5 μA, respectively. The maximum output power reached up to 8.31 μW. The electrical energy produced can directly supply small electronic devices such as LED. The breath sensor yields different output voltage signals under different breath strengths, indicating the ability to detect breathing in real‐time.

The composition of exhaled gas in human respiration also indirectly reflects their health status. In 2019, Wang et al. designed a breath sensor based on TENG that can harvest electric energy from the breath. More importantly, this self‐powered sensor can detect multiple respiratory parameters, such as respiratory frequency, the composition of exhaled gases, and human respiratory flow (Figure 4D).^[^ ^110^ ^]^ Ce‐doped ZnO‐polyaniline nanocomposites film and flexible PDMS with microstructure are used as main friction materials. Among them, the Ce‐doped ZnO‐PANI nanocomposites film was fabricated by hydrothermal and in‐situ polymerization methods. When the respiratory flow is fixed at 5 L/min, the real‐time output voltage signal of the breath sensor is about 0.88 V. The real‐time test results demonstrate that the TENG can be successfully driven by human respiration. It is worth noting that the sensor generates different voltage signals depending on the respiratory flow rate. Mainly, when exposed to an NH_3_ content range from 0.1 to 1 ppm, this self‐powered exhibits good NH_3_‐sensing performance, revealing a promising approach in disease screening associated with the detection of exhaled NH_3_ in humans breathing.

Zhu et al. designed a TENG with the help of machine learning to distinguish various volatile organic compounds (Figure 4E).^[^ ^111^ ^]^ This method exhibits the advantages of fast response, accurate quantization, and good selectivity. In this work, the multiswitched manipulation TENG was selected as a voltage source to provide high‐voltage output. The needle‐plate electrode leveraged the high voltage for plasma discharge in the gas chamber. Volatile organic compounds have different molecular structures and vibrational modes that affect their mid‐infrared response in the gas chamber. Therefore, the monitoring performances of the volatile organic compounds were boosted through the coupling of plasma and mid‐infrared absorption fingerprints. Eventually, the relationship between volatile organic compound species and their concentrations was visualized via machine learning aided method. When the gas mixed with ethanol flows in the gas chamber, a peak representing ethanol appears at the wavenumber of ∼2950 cm^−1^, and the intensity of this peak reflects the concentrations of ethanol.

### 3.3 Artificial sensory organs

Artificial sensory organs are aimed to mimic human sensory organs, which have shown significant importance in biomedical applications.^[^ ^117^ ^]^ Considerable progress has been made in artificial sensory organs in recent years through innovations in self‐powered technology based on NGs. These devices convert the energy (such as heat energy, acoustic energy, wind energy, and other mechanical stimulation) in the environment into electrical signals and analyze them, enables to gather the information in the external environment.^[^ ^22^, ^118^ ^]^

#### 3.3.1 Electronic skin

Electronic skin (e‐skin) is of significant meaning for next‐generation wearable electronics. In general, those devices can be divided into five categories depending on their work principle, including piezoresistive, capacitive, piezoelectric, TENG, and PENG. E‐skin based on NGs can continuously work without batteries, which has great potential application value in human‐computer interaction, national defense technology, etc.

**FIGURE 5:**
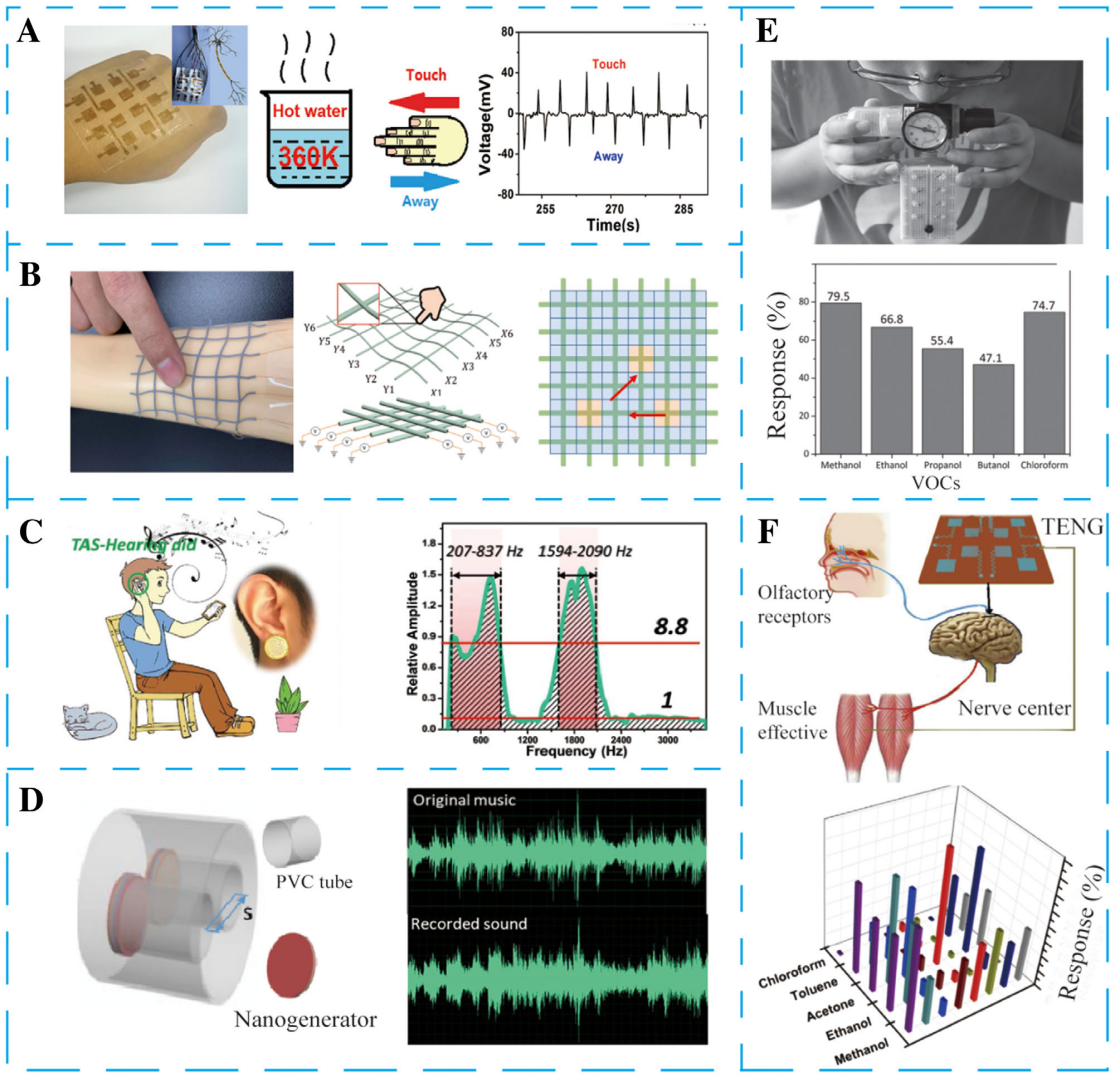
The fabricated NGs can be employed to be artificial sensory organs. (A) Single‐electrode PENG for bionic electric skin that can realize real‐time sensing of pressure integrating cold/heat sensing. Reproduced with permission.^[^ ^114^ ^]^ Copyright 2018, American Chemical Society. (B) 3D printed stretchable TENG for large‐scale and customized self‐powered electric skin. Reproduced with permission.^[^ ^115^ ^]^ Copyright 2021, Elsevier. (C) Self‐powered auditory sensor based on TENG for hearing aids. Reproduced with permission.^[^ ^102^ ^]^ Copyright 2018, American Association for the Advancement of Science. (D) Sound‐driven TENG for acoustic energy capturing and self‐powered multifunctional sensing. Reproduced with permission.^[^ ^66^ ^]^ Copyright 2019, Elsevier. (E) TENG based on triboelectrification/gas‐sensing coupling effect for flexible, smelling electronic skin. Reproduced with permission.^[^ ^116^ ^]^ Copyright 2019, Wiley‐VCH. (F) Flexible TENG linking to the brain for constructing artificial olfactory‐substitution perception‐behavior closed‐loop systems. Reproduced with permission.^[^ ^100^ ^]^ Copyright 2019, Elsevier

In 2018, Wang et al. proposed an e‐skin based on a single‐electrode PENG by electrospun PVDF nanofibers that can meet pressure and temperature sensing (Figure 5A).^[^ ^114^ ^]^ In the electrospinning process, PVDF films were spontaneously polarized, and the piezoelectric domain will be inclined to align along the external electric field. When the external force is exerted on the sensor or heated, the total spontaneous polarization within PVDF film changed, resulting in the production potential difference. In this case, external electronics will flow to the bottom and top electrodes to screen the potential difference, give rise to the production of an electrical signal. Therefore, two electric signals (from force and heat, respectively) can be acquired by a single unit simultaneously. When it is touched by something, the e‐skin will feel it. When the force disappears, it cannot feel it. Thus, the E‐skin can generate different electrical signals according to different pressures. Besides, it is easy to distinguish external pressure or temperature on the sensor from the voltage waveform. Finally, a large area flexible sensor array is formed by integrating multiple sensors to meet spatial resolution pressure and temperature detection requirements.

Usually, the intricate preparation processes and redundant structure blocks the application of the current e‐skin. In 2021, Chen et al. prepared stretchable elastic fibers by the 3D printing method (Figure 5B).^[^ ^115^ ^]^ This coaxial core‐shell structure (1 mm diameter) consists of the insulative sheath and conductive core, which can endure more than 300% strain and manifestation outstanding stretchable capacity. It is maybe attributed to that the interface between PTFE and flexible PDMS can be reoriented to avoid the occurrence and propagation of cracks. This structure and fabrication process proposed in this work removes the weaving process, thus enabling the entire fabrication process efficient and straightforward, and each fiber can be used as an independent triboelectric tactile sensing unit. In addition, this scalable smart textile has achieved large‐scale customized manufacturing. The function of e‐skin is realized through the preparation of a 6 × 6 array, which can quickly get the located information of the contact point by the acquisition of maximal output electrical signals in the array.

#### 3.3.2 Auditory sensors

Hearing impairment is one of the most serious diseases affecting the quality of human life. The backward technology limits the practical application of traditional cochlear implants, which forces researchers to seek fundamental improvements.^[^ ^119^ ^]^ In 2018, Guo et al. reported a self‐powered auditory sensor based on TENG for manufacturing an auditory system and successfully applied it in intelligent robotic (Figure 5C).^[^ ^102^ ^]^ The auditory sensor mainly consists of an FEP, acrylic, and Kapton membrane. With the self‐powered technology, the auditory sensor exhibits ultrahigh sensitivity and a broadband response range. The self‐powered auditory sensor can produce about 1.2 V output voltage under 100 dB sound pressure level and shows a resonant feature from the typical electric signal. Meanwhile, the output characteristics exhibit an exponential growth with the sound intensity. Even in a faint sound, the output of an electrical signal is still distinguished from noise. This sensor has the feature of easy‐fabrication and self‐powered, which indicates outstanding advantages of using self‐powered technology to construct the next generation of auditory systems for conquering the challenges in intelligent robotic.

In 2019, Chen et al. prepared an auditory sensor that can work stably in the frequency ranging from 20 to 1000 Hz (Figure 5D).^[^ ^66^ ^]^ This TENG with a unique structure comprises PVDF nanofibers film, two conductive fabrics, and a cylindrical PVC tube. This unique tubular structure achieved a high output performance by increasing the sound pressure and enhancing the external mechanical stimulation. The *V*_OC_, *I*_SC_, and instantaneous output power density can reach 400 V, 175 μA, and 7 W m^−2^ under the sound frequency of 170 Hz and the sound pressure of 115 dB, respectively. Furthermore, the self‐powered auditory sensor has been successfully used in velocity detection and acoustic source localization. For instance, when two auditory sensors are connected in parallel to capture sound energy, the sound source's distance and moving speed can be calculated according to the phase difference of two electrical signals. Simultaneously, the feasibility of acoustic sensors to record sound has also been manifested.

#### 3.3.3 Olfactory sensors

Since the invention of artificial olfactory sensors, the application of those in many different fields included medical diagnosis, has seen large development in these last two decades.^[^ ^120^ ^]^ In recent years, many advances have been made in self‐powered olfactory sensors. In 2016, Xue et al. reported a flexible e‐skin with olfactory functional basing on the triboelectrification/gas‐sensing coupling effect (Figure 5E).^[^ ^116^ ^]^ The conductive PANI films were served as both the functional layers and the electrode, and the PTFE film act as other friction material. The PTFE film with nanowire arrays is created via the ICP method to increase the surface charge density and effective contact area. The typical size of the device is 5 cm × 4 cm × 2 cm. The average value of *I*_SC_ and *V*_OC_ of the device is about ±1.21 μA and ±27 V under the gas flow rate is 12.5 m s^−1^ (imitate heavy human breath or blowing), respectively. Human activities can drive this self‐powered sensor, and the output characteristics are significantly dependent on the ambient atmosphere. Taking ethanol gas as an example, the detection limit of the olfactory sensor is 30 ppm, and the response is up to 66.8 against 210 ppm ethanol gas flow. This behavior can be ascribed to the PANI film can easily absorb the low‐molecular alcohols, affecting the output performance sensor.

In 2019, Zhong et al. proposed a self‐powered olfactory sensor using a flexible electronic smell detector linking to the brain like an olfactory epithelium (Figure 5F).^[^ ^100^ ^]^ The whole self‐powered sensor comprises PDMS/poly‐pyrrole sensing unit arrays. The olfactory sensor is connected to the brain and is driven by the harvested energy from body movement, forming a closed‐loop system. The PDMS with nanostructure film was served as both the friction materials and substrate. Cu network inlaid with Ppy acts as both other friction layer and gas sensing materials. Different volatile chemicals in the environment can react with specific Ppy derivatives inlaid in the Cu network, affecting the electrical output of a single sensor unit, thus realizing the function of olfactory sensors. When the device exposure to the different gas atmosphere, the sensing unit will generate a specific electrical signal and distinguish the information of gas species. Eventually, the produced triboelectric‐sensing signal is delivered to the mouse brain and exerts electrical stimulation for intelligent olfactory substitution.

## 4 SELF‐POWERED TECHNOLOGY FOR BIOMEDICAL APPLICATIONS

**FIGURE 6:**
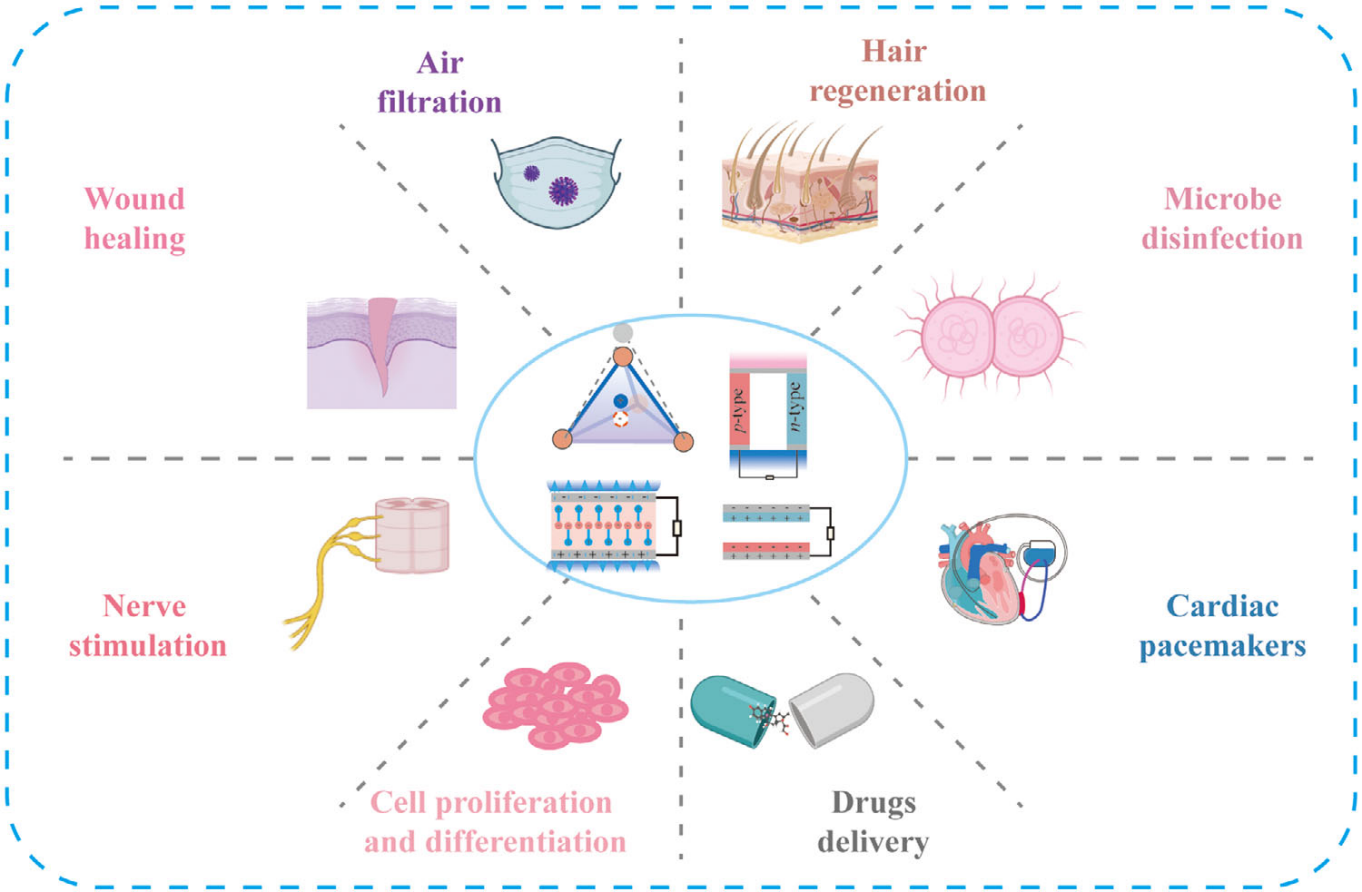
Self‐powered technology based on NGs is widely used in the biomedical field

As mentioned above, NGs, as independent sensors, convert biomechanical energy and thermal energy into electrical signals to reflect health information. Except for self‐powered sensors, numerous research has been devoted to finding other biomedical applications related to self‐powered technology, such as using electrical energy generated by NGs to stimulate biological tissues or power biomedical devices for achieving the purpose of medical application, which is of great significance in developing SPBEs.^[^ ^121^, ^122^, ^123^, ^124^ ^]^ Next, we classify and discuss the different applications, as shown in Figure 6.

### 4.1 Nerve stimulation

Nerve stimulation refers to using electric pulses that stimulate the specific nerve region for treatment, and it effectively alleviates various symptoms of neurological and psychiatric disorders.^[^ ^126^ ^]^ Traditional nerve stimulation therapy is usually accompanied by severe side effects, such as invasive surgeries, repeated treatment sessions, and infections. Meanwhile, implantable flexible electronic devices have attracted extensive attention in clinical applications in virtue of their excellent biocompatibility. Combining those two technologies provides some new cutting‐edge research fields, such as nerve stimulation devices based on self‐powered technology.

**FIGURE 7:**
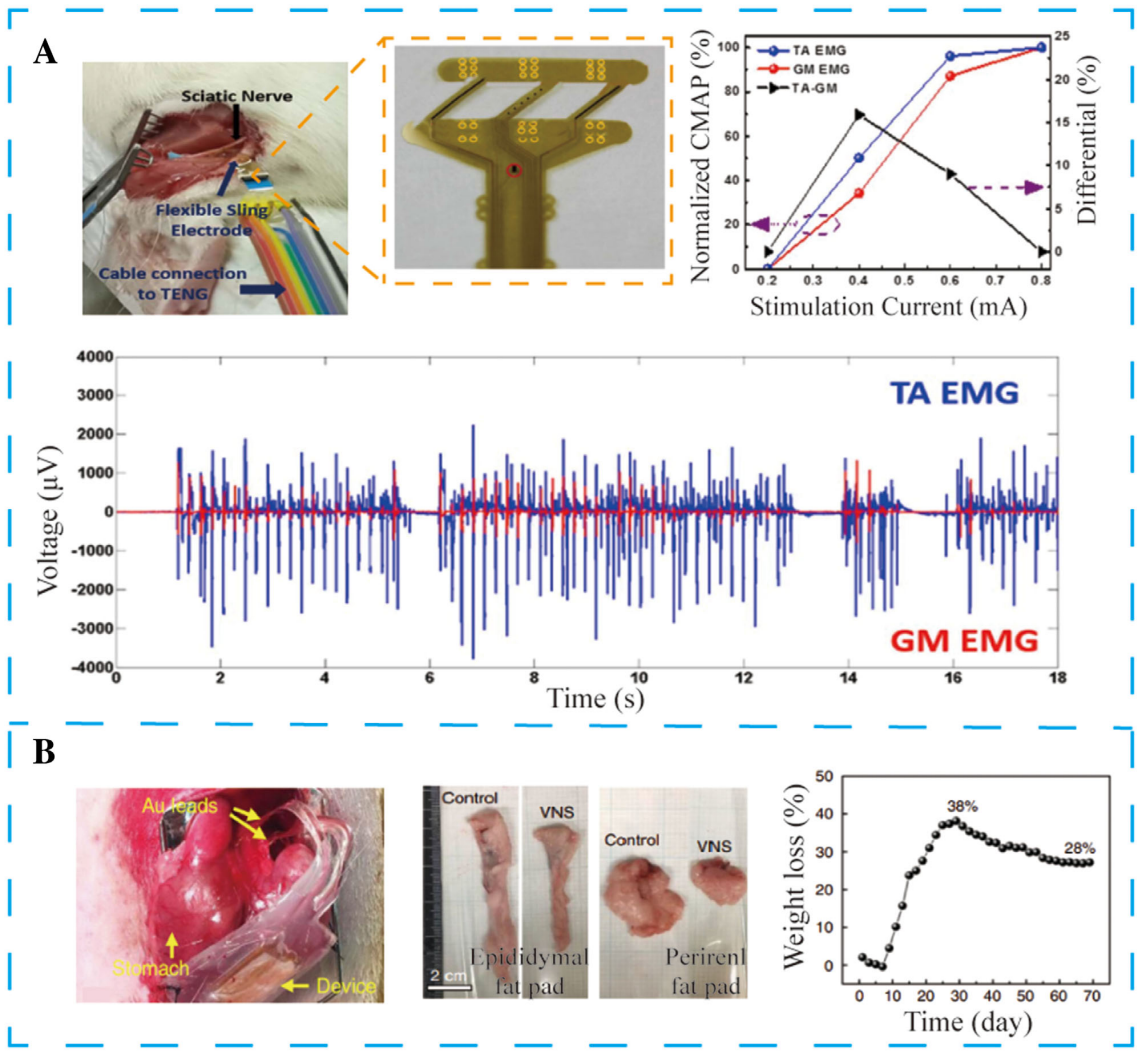
The fabricated NGs for in vivo neural stimulation system. (A) Stacked TENG with flexible and adjustable neural interfaces for sciatic neural stimulation. Reproduced with permission.^[^ ^26^ ^]^ Copyright 2017, Elsevier. (B) Self‐powered vagus stimulation system for reducing food intake and achieving weight control. Reproduced with permission.^[^ ^125^ ^]^ Copyright 2018, Nature Publishing Group

In 2017, Lee et al. designed a TENG with several units stacked together as a voltage source for neural stimulation through a tailoring neural interface (Figure 7A).^[^ ^26^ ^]^ Cu and flexible PDMS were acted as the primary functional materials. During the compression and release of the zigzag structure, the TENG composed of five units produced a maximum instantaneous output power of 51.8 μW. The optimized TENG is driven by muscle movement from the human body and delivers the electrical stimulation to a common peroneal nerve by neural interfaces constituted from a sling electrode to activate the anterior tibialis muscle. The flexible and implantable sling electrode has six active electrodes with each diameter of 0.1 mm and two circinate electrodes surrounding the nerve. With the aid of this flexible sling electrode, this TENG successfully achieves neural signal recording with different amplitudes.

In vivo vagus nerve stimulation (VNS) has excellent potential for treating some diseases, such as, regulating food intake for obesity treatment. In 2018, Yao et al. proposed a VNS device based on TENG without battery and automatically generates electrical stimulations to the vagus nerve (Figure 7B).^[^ ^125^ ^]^ The entire TENG was packaged by PDMS and ecoflex to ensure mechanical robustness, non‐cytotoxic, biocompatible, and flexibility of the whole system. The TENG can generate impressive high output performance, with a maximum output power of about 40 μW at an external load of 20 MΩ. During the distended and contraction of the stomach, the implantable TENG attached to the surface of the stomach generates electric energy to stimulate the vagal nerve to regulate food intake and finally achieve weight control. The generated maximum output voltage can reach about 0.8 mV. This treatment strategy has been successfully demonstrated in rats. The weight of the experimental rat has lost 38% within 15 days without a further rebound. This method is superior to all existing electrical stimulation approaches.

### 4.2 Tissue repairing and cell differentiation

Researchers have been of great interest in applying electrical stimulation in tissue repair and cell differentiation in the past decade.^[^ ^121^ ^]^ However, clinical electrical stimulation applications for tissue repairing and cell differentiation are still limited mainly by large‐sized extracorporeal devices, and patients may even need to be hospitalized.^[^ ^122^, ^127^, ^130^ ^]^ The recent innovation of self‐powered technology built a new avenue for producing periodic biphasic electric pulses by converting biomechanical energy. This distinctive capability makes NGs an ideal choice for generating real‐time and sustainable electrical stimulations.

#### 4.2.1 Wound healing

**FIGURE 8:**
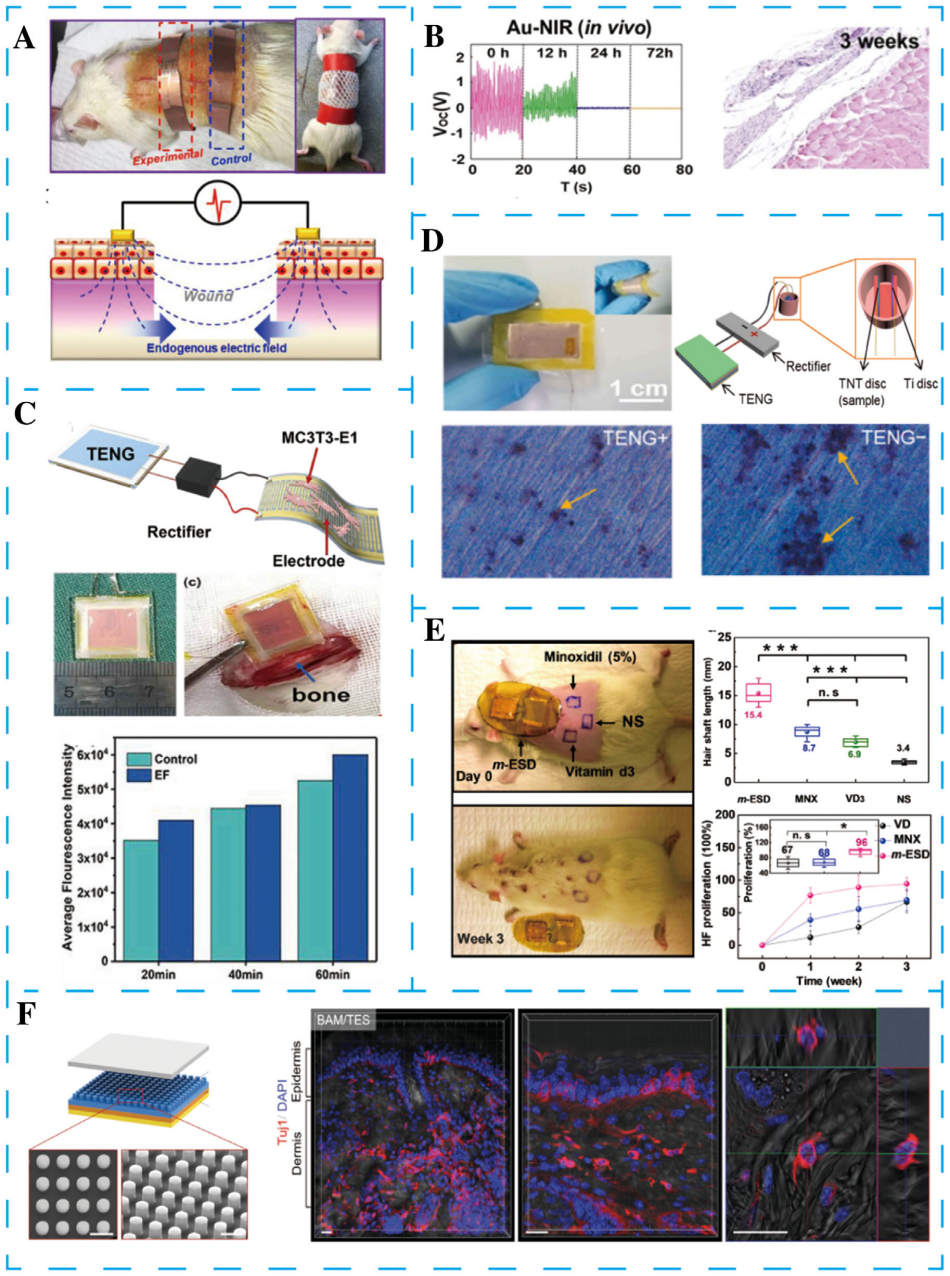
Various NGs have been applied in tissue repairing and cell differentiation. (A) TENG produces the discrete alternative electric fields produced for accelerating wound healing. Reproduced with permission.^[^ ^127^ ^]^ Copyright 2018, American Chemical Society. (B) Photothermally tunable biodegradable implantable TENG for tissue repairing. Reproduced with permission.^[^ ^128^ ^]^ Copyright 2018, Elsevier. (C) Self‐powered implantable electrical stimulator based on TENG for osteoblasts’ proliferation and differentiation. Reproduced with permission.^[^ ^129^ ^]^ Copyright 2019, Elsevier. (D) Implantable TENG for building the stable and long‐term effective negatively charged implant surface. Reproduced with permission.^[^ ^130^ ^]^ Copyright 2019, Elsevier. (E) Self‐activated electrical stimulation system based on wearable pulse generator for effective hair regeneration. Reproduced with permission.^[^ ^131^ ^]^ Copyright 2019, American Chemical Society. (F) Periodic biphasic pulse‐like currents generated from TENG for in vivo reprogramming of fibroblasts to functional neuronal cells. Reproduced with permission.^[^ ^132^ ^]^ Copyright 2016, Wiley‐VCH

Slow or unhealed skin wounds can lead to long‐term physical and mental pain and enormous health care costs. In 2018, Long et al. designed a wearable TENG device that can produce small electrical pulses to significantly accelerated skin wound healing (Figure 8A).^[^ ^127^ ^]^ When the TENG is wrapped around the chest area of a rat, the overlapping area change produced by breathing would drive charge flow between two dressing electrodes and, thus, generate an electrical signal. When the rat was under regular activity, the output voltage reached the highest level of about 2.2 V. After two days under regular training, the wound region covered by the electrode in the experimental groups showed a nearly complete recovery. The further study confirmed that the small discrete electric field generated by TENG guided and promoted wound recovery along the direction of the electric field. A series of in vitro experiment results revealed that the therapeutic effects were related to the electric field, directing fibroblast proliferation, migration, and transdifferentiation.

Biodegradable medical electronic devices can avoid surgery to remove those devices after achieving their goal, which has an essential significance for short‐term treatment. In 2018, Li et al. fabricated a serial of biodegradable TENGs for accelerating wound healing (Figure 8B).^[^ ^128^ ^]^ The as‐fabricated biodegradable TENGs consist of friction layers, back electrodes, Au‐doped poly L‐lactide‐*co*‐glycolide film, and encapsulation layer. The biodegradable friction layers were fabricated by a template method to enlarge the contact area and the spacer between them was omitted. The degradation process in vivo of those TENGs can be effectively tuned by Au nanorods, attributed to the Au nanorods responding to the near‐infrared light sensitively. Under near‐infrared light, the device gradually degraded into small components in 14 days. The electrical field was delivered from TENG to the L929 cells by the electrode for accelerating wound healing. By comparison with the control group, wound healing was more efficient than traditional DC stimulation. There may be two reasons for these results: the alternating electric field promoted cell migration from the two margins. The second reason is the accelerated cell proliferation promotes wound healing.

#### 4.2.2 Osteoblasts proliferation and differentiation

Bone remodeling or orthodontic treatment is usually a long‐term process.^[^ ^122^ ^]^ The invention of NGs may provide an effective method to promote the proliferation and differentiation of osteoblasts. In 2019, Tian et al. designed a self‐powered flexible and implantable electrical stimulator based on TENG for cells differentiation (Figure 8C).^[^ ^129^ ^]^ A PTFE film with nanostructured acted as a mani friction layer. The flexible TENG was implanted into the surface of the Sprague Dawley rat's femur and could convert the mechanical energy from the daily activities into electric energy. During the experiment, the number and differentiation level of MC3T3‐E1 cells was significantly higher than that of the control group. These results lay a foundation for the future use of TENG to stimulate osteoblasts proliferation and differentiation.

Shi et al. used TENG to capture daily human movement for construction stable and long‐term effective negative charge implant surface, which effectively inhibit bacterial adhesion and reduce the ratio of live/dead bacteria to form and mature biofilm (Figure 8D).^[^ ^130^ ^]^ PTFE with nanorods and the anodized Ti foil with nanotubes were used as triboelectric materials. The other face of Ti foil without anodization and a copper film prepared by magnetron sputtered were served as electrodes. Subsequently, the naked TENG with the size of 1.6 cm × 1 cm was encapsulated in PDMS, which generated a *V*_OC_ of 12 V. The TENG is contacted to the discs through a rectifier to eradicate bacteria, thus promoting the MC3T3‐E1 proliferation. The experimental result shown the number of nodules in the TENG‐group was much greater than that of other control groups, reveals the negatively charged surface is more conducive to promoting MC3T3‐E1 cells to differentiate into osteoblasts, which may be due to the limitation of the growth of bacteria in the micro alkaline environment.^[^ ^133^ ^]^

#### 4.2.3 Hair regeneration

Hair loss caused by a lack of growth factors and a disorder of the hair cycle is a common and upsetting disease. In 2019, Yao et al. proposed an effective method for promoting hair regeneration using a new wearable electrical stimulation device (Figure 8E).^[^ ^131^ ^]^ The electrical stimulation device consists of TENG and interdigitated electrodes. The whole device is driven by daily motions and generated voltage signals. The produced electrical energy was directly used to stimulate hair regeneration through the interdigitated dressing electrodes. The stimulation device promoted hair transformation from the telogen stage to the anagen stage and thereby accelerated hair growth rate. Importantly, this method could conquer the genetic keratin disorder and achieve effective hair regeneration on a nude rat, which may be due to the paracrine mechanism of hair growth factors.^[^ ^134^ ^]^

#### 4.2.4 Neuronal cell differentiation and oriented growth

Some researchers have reported that endogenous electrical signals play an essential role in the process of neural cell differentiation and maturation, such as promoting the growth and differentiation.^[^ ^135^, ^136^ ^]^ In 2016, Jin et al. applied the TENG as a voltage source to produce periodic biphasic pulse‐like current signals to promoted the regeneration of neuronal cells (Figure 8F).^[^ ^132^ ^]^ Al and PTFE with the micro‐pillar structure were chosen as the TENG's top and bottom friction layer, which will exhibit an impressive output performance due to the huge electronegativity difference between them. The TENG can produce controllable, naturally occurring electrical signals for promoting cell conversion. In the process of cell conversion, the stimulation device is connected to the highly conductive Ti‐coated cell culture substrate to achieve the direct conversion of primary mouse embryonic fibroblasts to induced neuronal cells. The results showed that this new electrical stimulation method effectively accelerated the transformation of nerve cells. This phenomenon may be due to the increase of intracellular Ca^2+^ ions concentration after electrical stimulation. Ca^2+^ plays a crucial part in neuronal growth, which is involved in the induction of various cellular functions and genes related to cell maturation.

Zheng et al. proposed a TENG for short‐term in vivo biomechanical energy harvesting (Figure 8G).^[^ ^137^ ^]^ Two of the biodegradable polymers layers were acted as primary friction layers. Finally, the entire device was embed in biodegradable polymers layers to prevent damage by the surrounding environment. Thus, the TENG can be degraded and resorbed by the animal body without any adverse impact. The TENG was connected to the complementary patterned copper electrode networks through a rectifying bridge. The corresponding electric field between the electrode was 10 V/mm. When the TENG was implanted in the subdermal region of the backs of rats, most of the electrically stimulated neurons were well oriented, and the cytoskeleton of neurons was along the electric field direction. The electric field produced by TENG has a significant effect on the arrangement of neurons, which is very important for nerve repair.

### 4.3 Pacemakers

**FIGURE 9:**
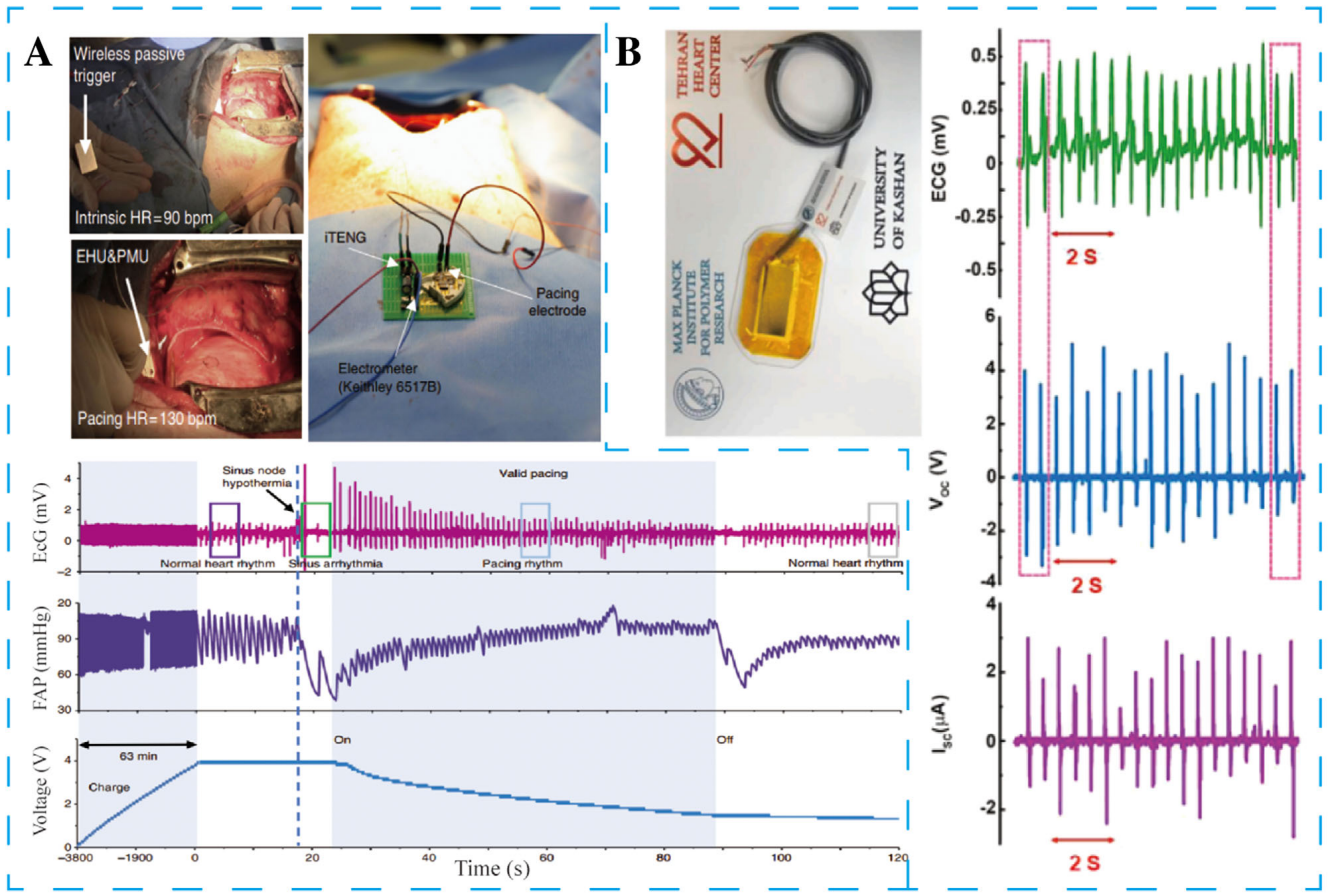
The NGs can be utilized to be cardiac pacemakers. (A) Symbiotic pacemaker based on TENG successfully corrects sinus arrhythmia and prevents deterioration. Reproduced with permission.^[^ ^6^ ^]^ Copyright 2019, Nature Publishing Group. (B) Implantable PENG harvests electrical energy from the cardiac motions of the left ventricle to power the cardiac pacemaker. Reproduced with permission.^[^ ^138^ ^]^ Copyright 2021, Elsevier

Cardiac pacemakers can remarkably prolong patients' lives with sick sinus syndrome or heart block by stimulating cardiac muscle and regulating heartbeat with an electric pulse.^[^ ^139^ ^]^ Self‐powered cardiac pacemakers are powered by implantable NGs, which can break down the restriction of the limited life of commercial pacemakers.^[^ ^8^, ^123^ ^]^ In 2019, Ouyang et al. proposed an implantable symbiotic pacemaker based on a TENG (Figure 9A).^[^ ^6^ ^]^ The symbiotic pacemaker consisted of TENG, energy management unit, and pacemaker. The output characteristics of the packaged device avoid the adverse effects of wet conditions. During the animal experiment, the TENG was connected to a capacitor via a rectifier. It is noteworthy that the energy capture from each heart pulse cycle is higher than the required endocardial pacing threshold energy. When the switch of the energy management was turn on, sinus arrhythmia was converted to pacing rhythm. Simultaneously, the systolic blood pressure declined fast from ∼100 to ∼60 mmHg owing to the fast pacing by the symbiotic pacemaker. The above results indicating the symbiotic pacemaker adjusted sinus arrhythmia and prevented further deteriorating conditions.

Compared with TENG, PENG is more adept in converting mechanical stimulation into electrical energy in a narrow space, especially in vivo, and this ability is independent of time and location.^[^ ^13^, ^19^ ^]^ In 2021, Azimi et al. proposed a pacemaker that is driven by a biocompatible and flexible PENG from cardiac motions (Figure 9B).^[^ ^138^ ^]^ Considering the left ventricles have the largest motion amplitude than right ventricles, the PENG was sutured on the pericardium, facing the lateral left ventricles. As a result, the PENG has produced voltage signals as high as 3.9 V, synchronous with the heartbeat. Meanwhile, the harvested energy from the heartbeats is stored in a 100 μF capacitor by a rectifier, which is sufficient to power a typical commercial pacemaker. Eventually, the captured energy powers a commercial pacemaker, successfully realizing a self‐powered pacemaker device.

### 4.4 Drugs delivery

**FIGURE 10:**
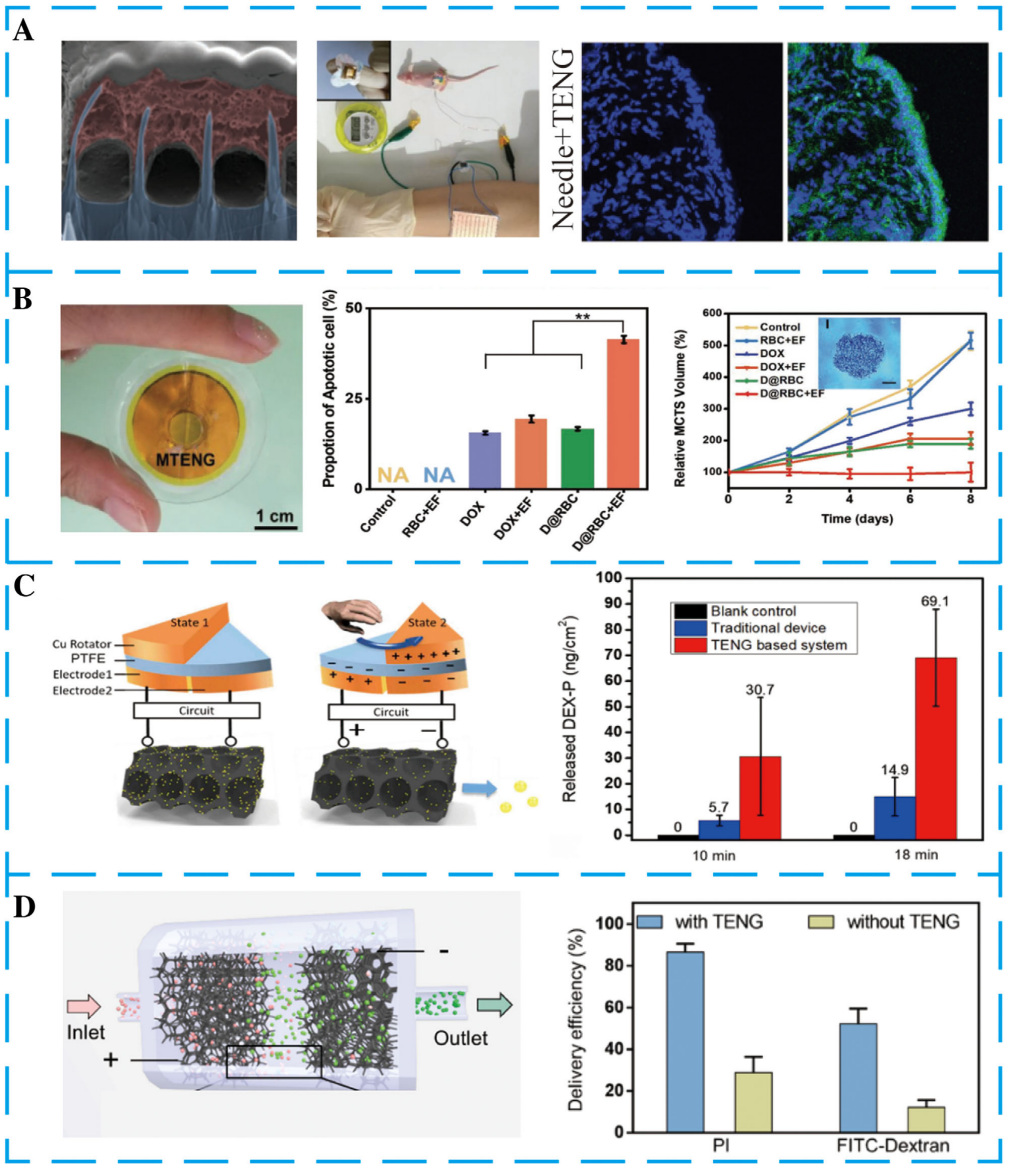
The application of NGs for drug delivery. (A) Self‐powered electroporation system for intracellular high‐efficiency drug delivery. Reproduced with permission.^[^ ^140^ ^]^ Copyright 2019, Wiley‐VCH. (B) A nanogenerator‐controlled drug delivery system for eliminating carcinomatous cells. Reproduced with permission.^[^ ^141^ ^]^ Copyright 2019, Wiley‐VCH. (C) An on‐demand, self‐powered transdermal drug delivery system. Reproduced with permission.^[^ ^142^ ^]^ Copyright 2019, Elsevier. (D) TENG‐driven high‐throughput and self‐powered electroporation system for drug delivery. Reproduced with permission.^[^ ^143^ ^]^ Copyright 2020, American Chemical Society

Non‐destructive, efficient, and on‐demand drug delivery for disease treatment has great significance yet presents major challenges. Using ubiquitous mechanical energy to power electroporation is a promising method to realize self‐powered drug delivery.^[^ ^142^, ^144^ ^]^ In 2019, Liu et al. designed in vivo electroporation drug delivery powered by biomechanical energy (Figure 10A).^[^ ^140^ ^]^ The hand‐powered TENG produces a continuous pulsed electrical voltage between two electrodes for electroporation. This kind of silicon nanoneedle array can reduce the applied voltage and damage cells' concomitant damage. Simulation results prove the notable enhancement in the electrical field at the tip of each nanoneedle. When a voltage of 20 V is applied, the corresponding electric field is as high as 2800 V cm^−1^. The fluorescent images of tissue cryosections confirmed that the drug delivery distance under TENG electrical stimulation was deeper than that without electrical stimulation and beyond the height of the nanoneedle. This may be attributed to the fact that the cell will open transient pores during electroporation due to the local electric field at the tip of the nanoneedle. These results demonstrated the feasibility of using TENG to convert biomechanical energy into electrical energy for drug delivery in vivo.

In drug delivery, red blood cells (RBC) are one of the most promising vehicles. Zhao et al. designed a drug delivery system based on TENG by electric field controlled (Figure 10B).^[^ ^141^ ^]^ To overcome the detach tendency between two friction layers gradually decreases, a pair of small magnets were installed on the backside of friction layers to separate them. This TENG with a unique structural design can exhibit excellent and stable output characteristics after encapsulation. The output voltage of the magnet‐TENG is up to 70 V after implantation, the equivalent electric field generated is 4 kV cm^−1^. Under the electric stimulation of TENG, the release of doxorubicin‐loaded in RBC dramatically increases and decreases to the normal state after electrical stimulation. Those results indicating the eliminating efficiency of cancer cells at a low drug dosage is a significant improvement by the accurate and effective release of the doxorubicin.

Ouyang et al. integrated TENG and a homemade power management unit to achieve a self‐powered drug delivery system (Figure 10C).^[^ ^142^ ^]^ This radial‐arrayed rotary TENG can convert the biomechanical energy into electrical energy to power the management circuit. The homemade power management circuit can stabilize released electrical energy for on‐demand drug release. Once an electric field is formed on the skin surface, the charged drug molecules cross the stratum corneum by electrophoresis and electroosmosis effects. It is worth noting that the drug release rate increased with the duration of the releasing stimulus.

Although tremendous progress has been made in drug delivery, the low‐throughput and inevitable cell injury have severely blocked the development. In 2020, Liu et al. proposed a high‐throughput electroporation system based on a multilayered TENG (Figure 10D).^[^ ^143^ ^]^ Among them, this multilayered TENG can generate *V*_OC_ and the transferred charge was 220 V and 780 nC under a mechanical linear motor at the frequency of 2.5 Hz, respectively. When the voltage produced by the TENG is applied to the electrodes, an electrical field for cell electroporation will be created. Notably, the anode microfoam modifies with one‐dimension (1D) Ag nanowires for generating a local electric field, which could reduce cell damage, especially at the sharp nanoscaled tips. This electroporation system exhibits high cell throughput and delivery efficiency, indicating the feasibility of the electroporation system based on TENG for high‐throughput intracellular delivery.

### 4.5 Healthcare

#### 4.5.1 Microbe disinfection

**FIGURE 11:**
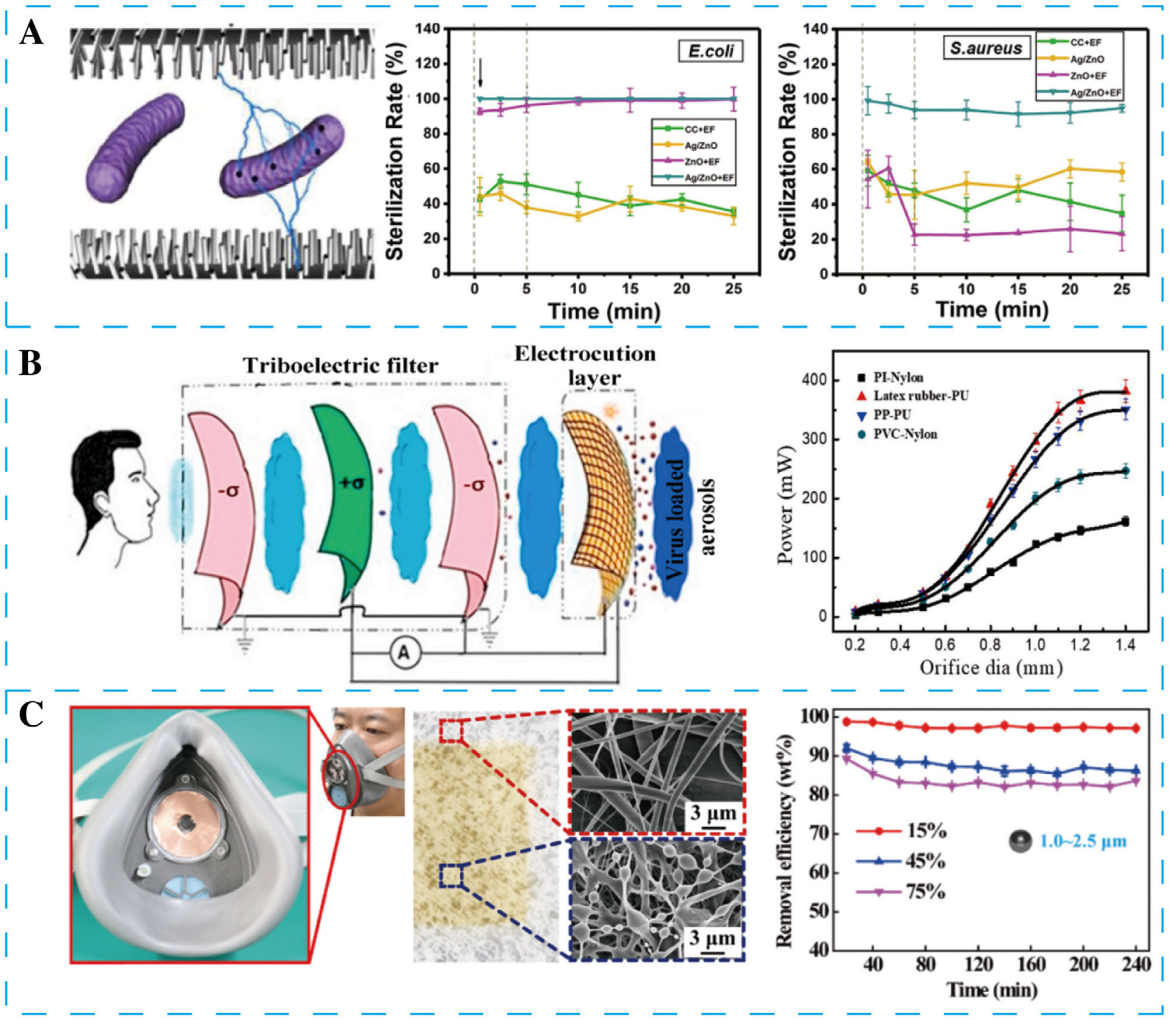
Various NGs have been used in healthcare applications. (A) A self‐powered sterilization system based on ball‐in‐ball TENG for instant and sustainable antibacterial. Reproduced with permission.^[^ ^124^ ^]^ Copyright 2017, Elsevier. (B) A face mask based on TENG may use for virus filtration. Reproduced with permission.^[^ ^145^ ^]^ Copyright 2021, Elsevier. (C) Self‐powered electrostatic adsorption mask based on TENG for ultrafine particulates filtration within the air. Reproduced with permission.^[^ ^146^ ^]^ Copyright 2018, American Chemical Society

Electrical interactions between bacteria and the environment are delicate and essential.^[^ ^124^ ^]^ In 2018, Tian et al. developed a self‐powered water sterilization system based on a wave‐driven TENG and two nano brush electrodes (Figure 11A).^[^ ^124^ ^]^ In the sterilization process, the pulse electrical signals were applied in two electrodes with Ag/ZnO nano brush. Many electrons are first gathered in Ag nanoparticles in the disinfection process and then transferred in ZnO nanowires. Once the electric field is withdrawn, the stored electrons are released to reactive oxygen species, which leads to bacteria lysis and death. The system exhibits an immediate and sustainable high‐efficient sterilization effect on microorganisms in natural river water. The sterilization rate of *Escherichia coli* and *Staphylococcus aureus* up to 100% and 95%, respectively. After electric field withdrawal, the colony‐forming units stopped growing for the next 20 min.

The face mask has played a major role in fighting the SARS‐CoV‐2. In 2021, Ghatak et al. presented a face mask based on TENG to filtrate and deactivate SARS‐CoV‐2 (Figure 11B).^[^ ^145^ ^]^ The face mask comprises multilayer filters film, including the inner three layers are used as triboelectric layers, and the outer one is used as an electric cutting layer. A positive friction layer sandwiched between two negative friction layers generates an output voltage while breathing. The generated triboelectric charges are stored in a capacitor for disinfection of incoming and outgoing virus‐containing aerogels. Therefore, virus particles may be disinfected by electrocution, but the results have yet to be verified.

#### 4.5.2 Air filtration

With the rapid development of industrialization, particulate pollution is severely affecting the life quality of people, especially in developing areas. Traditional masks have limited removal efficiency for particles with a size range of 10–1000 nm, which seriously endangers human health. In 2018, Liu et al. reported a novel self‐powered electrostatic adsorption face mask using a TENG and PVDF nanofiber film (Figure 11C).^[^ ^146^ ^]^ PVDF nanofiber film and Cu film act as friction materials and electrodes, respectively. The TENG is fixed into the inlet of a face mask. When the air mixed with the particulates flows through the mask, the coarse particulates are filtered out by the PVDF nanofiber film due to the physical filtration mechanism. The remaining fine and especially ultrafine charged particles are adsorbed by the negative or positive area of the PVDF nanofiber film. The PVDF nanofiber film charges the uncharged particles through the contact electrification mechanism and adsorption by nanofiber film. Eventually, all of the particles are absorbed into the mask.

## 5 CHALLENGES AND FUTURE PERSPECTIVES

SPBEs have been speedily developed in recent years in virtue of their exceptional merit in the medical field, which will significantly impact the healthcare industry. Despite most SPBEs have their distinctive advantages, considering that as an emerging field, there are still some noticeable blocks that need to be solved urgently.

### 5.1 Device packaging and biocompatibility

To date, various NGs have been acted as energy harvesters and independent sensors in the biomedical field. Considering that SPBEs are usually in close contact with the human body, the packaging and biocompatibility of the SPBEs must arouse our attention. Given the complex and irregular physiological activities of the human body, flexible packaging can make the device bear all kinds of deformation without damage. On the other hand, implantable devices to be immersed in body fluids for long periods. Therefore, tight packaging can prevent body fluids from leaking into the internal structure and ensure that the device's performance is not affected by the external environment.

Secondly, any application contacted with skin or tissues will require non‐biotoxicity, which can effectively avoid adverse immune reactions. Finally, attention should be paid to the fact that the performance of the encapsulated SPBEs will generally decline due to the packaging layer will affect the stress transfer or deformation, especially the SPBEs that depend on the conversion of biomechanical energy into electrical energy. Therefore, it is necessary to strictly control the thickness of the packaging layer to maintain the sensitivity of SPBEs to biomechanical movement.

Over the past years, many reports have considered flexible polymer with biocompatibility as the future of ideal encapsulation materials.^[^ ^147^, ^148^ ^]^ Among many packaging materials, flexible polymers such as PDMS and polyimide (PI) films are widely used in wearable devices and implantable devices since it has both flexible, and biocompatible. Although the PI has not been approved by Food and Drug Administration, researchers have evaluated it at the Lawrence Livermore National Lab using ISO10993 standards and being USP Class VI compliant.^[^ ^149^ ^]^ PI was tested in complex conditions for 7 months, indicating that PI has outstanding biocompatibility in the long‐term application.^[^ ^150^, ^151^ ^]^ At the same time, it is also an excellent pathway to achieve this goal using biocompatible metal oxides. An Al_2_O_3_ film (40 nm) was deposited onto the PDMS by atomic layer deposition, forming a dense metal oxide protective layer.^[^ ^152^ ^]^

### 5.2 Miniaturization

The miniaturization of SPBE is also a problem that cannot be ignored. Miniaturization of the SPBEs allows them to contact more closely with the skin and muscle. For instance, self‐powered pulse sensors are usually fixed at the wrist of the human to real‐time monitoring the pulse. The bulky size of the pulse sensors will affect the stability and disturb the test results due to uneven stress distribution. On the other hand, large‐sized implantable SPBEs often require major surgery for implantation. Therefore, the size of the implantable SPBEs is closely related to the patient's quality of life. For instance, in terms of self‐powered cardiac pacemakers, the size and weight device should be controlled within 16 × 16 mm and 6 g.^[^ ^153^ ^]^ Importantly, there is evidence that, regardless of the rigidity of the selected material at the cellular level, miniaturized devices may still prevent immune responses.^[^ ^154^ ^]^ Although SPBE has not yet reached the size of cells, it will be promising.

### 5.3 Long‐term stability

With the advancement of the preparation process, the flexibility and fracture tolerance of NG has been greatly enhanced. However, one of the most critical requirements for SPBEs development is long‐term stability, especially for implantable devices. This feature will save patients from unnecessary pain and financial burden. In general, NGs maintain excellent adhesion to adjacent skin so that they exhibit a stable electrical output throughout the test period. As for implantable devices, it is more difficult to keep them in intimate contact with organs or muscles. Early reports suggest that suturing is an effective solution to this problem. Han et al. sutured NG into the pericardium to collect the energy from the beating heart, eventually realizing a symbiotic cardiac pacemaker.^[^ ^6^ ^]^ If this method of stitching is used, the miniaturization of the device must also be taken into account. Therefore, for implantable devices, we have to find a balance between miniaturization and long‐term stability, depending on the case.

### 5.4 Flexible power management circuit

Although Xi et al. reported a universal power management circuit with the store, adjust, and stabilize the electrical energy, the rigid substrate of the circuit cannot form a close interface with human tissues, which is an urgent problem.^[^ ^155^ ^]^ Particularly, implantable SPBEs must be implanted or fixed on soft human tissues or organs in a stable and close manner. Reducing the mechanical mismatch of the interface between SPBE and the organism can effectively avoid the adverse immune response during the chronic implantation process, thereby improving biological safety. Meanwhile, challenge breeds opportunity. With the evolution of integration technology, the power management circuit within the SPBE of the future will become smaller and more powerful than ever, and even become flexible and bendable. We may be able to pursue further optimization of the structural design of electronic devices first. For example, researchers can first focus on developing biocompatible material with low Young's modulus acted as a circuit substrate, effectively eliminating the mechanical mismatch at the interface between the energy management circuit and organism.^[^ ^156^ ^]^

### 5.5 Future perspectives

So far, self‐powered technology has made its mark in biomedical applications. Unfortunately, there is still a big challenge to meet the practical applications in the biomedical field. Personally, the main reason for limiting its development, in addition to the four points mentioned above, is to improve the output performance and reduce the internal impedance of the NGs is also the key. Improving the output performance of the NGs can not only ensure the stable working of the self‐powered device but also power more components to achieve rich features. Multi‐functional characteristics are also the goal of self‐powered technology has been pursued. Self‐powered devices with multi‐functional characteristics have a wider range of applications, which will accelerate the development of SPBEs. Second, reducing the internal impedance of the NGs can effectively match NGs with external electronics and improve the energy conversion efficiency. Although it has been reported that the internal impedance of NGs has been effectively reduced by well‐designed circuits, for implantable electronic devices, issues such as miniaturization, flexibility, and biocompatibility need to be taken into account simultaneously. In turn, these issues are intertwined and mutually constrained. Finding a balance between them based on practical applications may be the key to the future development of SPBE.

## 6 CONCLUSION

Proper use of biomechanical energy captured from biological and human activities has important practical significance for medical diagnosis and treatment. Various technologies have been proposed to convert these energies to electrical energy in past decades, such as NGs. Using these energies can help design SPBEs with distinctive functional characteristics and benefit for improve quality of life, such as pulse sensors and symbiotic cardiac pacemakers. In the future, the widespread applications of SPBEs will have a significant impact on our daily life, from healthcare to disease treatment.

In this review, we first introduce four kinds of nanogenerators according to different energy harvesting principles to deepen the understanding of self‐powered technology. Afterward, according to the different working characteristics, SPBEs can be classified into two main categories: The first is that NGs, as an independent sensor, directly convert the energy generated by physiological activities into electrical signals. Finally, the patient's health status is judged through the analysis of electrical signals. Besides self‐powered sensors, another research direction is to directly use the electrical energy generated by NGs to achieve medical applications, such as tissue repairing, drug delivery, and microbial sterilization. In addition, the underlying challenges and development prospects of SPBEs are discussed. In the future, as the device design and data handling technologies will be further tailored to specialized needs, SPBEs are likely to be adopted as mainstream solutions in clinical diagnosis and treatment.

## CONFLICT OF INTEREST

The authors declare no conflict ofinterest.

## Data Availability

The data that support the plots within this paper and other findings of this study are available from the corresponding author upon reasonable request. Source data are provided with this paper.
